# Proteome Analysis Reveals the Conidial Surface Protein CcpA Essential for Virulence of the Pathogenic Fungus *Aspergillus fumigatus*

**DOI:** 10.1128/mBio.01557-18

**Published:** 2018-10-02

**Authors:** Vera Voltersen, Matthew G. Blango, Sahra Herrmann, Franziska Schmidt, Thorsten Heinekamp, Maria Strassburger, Thomas Krüger, Petra Bacher, Jasmin Lother, Esther Weiss, Kerstin Hünniger, Hong Liu, Peter Hortschansky, Alexander Scheffold, Jürgen Löffler, Sven Krappmann, Sandor Nietzsche, Oliver Kurzai, Hermann Einsele, Olaf Kniemeyer, Scott G. Filler, Utz Reichard, Axel A. Brakhage

**Affiliations:** a Department of Molecular and Applied Microbiology, Leibniz Institute for Natural Product Research and Infection Biology (HKI), Jena, Germany; b Department of Microbiology and Molecular Biology, Institute of Microbiology, Friedrich Schiller University Jena, Jena, Germany; c Institute for Medical Microbiology, University Medical Center, Göttingen, Germany; d Transfer Group Anti-infectives, Leibniz Institute for Natural Product Research and Infection Biology (HKI), Jena, Germany; e Institute of Clinical Molecular Biology, Christian-Albrechts University Kiel, Kiel, Germany; f Institute of Immunology, Christian-Albrechts University Kiel & Universitätsklinik Schleswig-Holstein, Kiel, Germany; g Medical Clinic and Policlinic II, University Clinic Würzburg, Würzburg, Germany; h Institute for Hygiene and Microbiology, University of Würzburg, Würzburg, Germany; i Septomics Research Center, Leibniz Institute for Natural Product Research and Infection Biology (HKI), Jena, Germany; j Division of Infectious Diseases, Los Angeles Biomedical Research Institute at Harbor-UCLA Medical Center, Torrance, California, USA; k David Geffen School of Medicine at University of California, Los Angeles, Los Angeles, California, USA; l Microbiology Institute-Clinical Microbiology, Immunology and Hygiene, University Hospital Erlangen and Friedrich-Alexander University of Erlangen-Nürnberg, Erlangen, Germany; m Center for Electron Microscopy, Jena University Hospital, Jena, Germany; n Internal Medicine II, University Hospital Würzburg, Würzburg, Germany; Karlsruhe Institute of Technology (KIT)

**Keywords:** *Aspergillus fumigatus*, CcpA, T cells, dendritic cell, epithelial cell, immune response, mass spectrometry, neutrophil, resting conidia, surface proteome, swollen conidia, trypsin shaving

## Abstract

Aspergillus fumigatus is a common airborne fungal pathogen of humans and a significant source of mortality in immunocompromised individuals. Here, we provide the most extensive cell wall proteome profiling to date of A. fumigatus resting conidia, the fungal morphotype pertinent to first contact with the host. Using liquid chromatography-tandem mass spectrometry (LC-MS/MS), we identified proteins within the conidial cell wall by hydrogen-fluoride (HF)–pyridine extraction and proteins exposed on the surface using a trypsin-shaving approach. One protein, designated conidial cell wall protein A (CcpA), was identified by both methods and was found to be nearly as abundant as hydrophobic rodlet layer-forming protein RodA. CcpA, an amphiphilic protein, like RodA, peaks in expression during sporulation on resting conidia. Despite high cell wall abundance, the cell surface structure of Δ*ccpA* resting conidia appeared normal. However, trypsin shaving of Δ*ccpA* conidia revealed novel surface-exposed proteins not detected on conidia of the wild-type strain. Interestingly, the presence of swollen Δ*ccpA* conidia led to higher activation of neutrophils and dendritic cells than was seen with wild-type conidia and caused significantly less damage to epithelial cells *in vitro*. In addition, virulence was highly attenuated when cortisone-treated, immunosuppressed mice were infected with Δ*ccpA* conidia. CcpA-specific memory T cell responses were detectable in healthy human donors naturally exposed to A. fumigatus conidia, suggesting a role for CcpA as a structural protein impacting conidial immunogenicity rather than possessing a protein-intrinsic immunosuppressive effect. Together, these data suggest that CcpA serves as a conidial stealth protein by altering the conidial surface structure to minimize innate immune recognition.

## Abstract (precis)

The mammalian immune system relies on recognition of pathogen surface antigens for targeting and clearance. In the absence of immune evasion strategies, pathogen clearance is rapid. In the case of Aspergillus fumigatus, the successful fungus must avoid phagocytosis in the lung to establish invasive infection. In healthy individuals, fungal spores are cleared by immune cells; however, in immunocompromised patients, clearance mechanisms are impaired. Here, using proteome analyses, we identified CcpA as an important fungal spore protein involved in pathogenesis. A. fumigatus lacking CcpA was more susceptible to immune recognition and prompt eradication and, consequently, exhibited drastically attenuated virulence. In infection studies, CcpA was required for virulence in infected immunocompromised mice, suggesting that it could be used as a possible immunotherapeutic or diagnostic target in the future. In summary, our report adds a protein to the list of those known to be critical to the complex fungal spore surface environment and, more importantly, identifies a protein important for conidial immunogenicity during infection.

## INTRODUCTION

The saprophytic fungus Aspergillus fumigatus is among the most important human-pathogenic fungi. A. fumigatus can cause serious invasive lung infections, designated invasive aspergillosis (IA), in patients immunocompromised by hematologic malignancies, organ transplantation, or genetic immunodeficiency (1–4). As a consequence of the increasing number of patients with a compromised immune system, the incidence of IA has risen steadily over the last decades. Specific diagnostics and treatment options for IA are limited, resulting in high (30% to 95%) mortality rates (5). It is estimated that 200,000 cases of IA occur each year worldwide. Despite this tremendous health care burden, the molecular pathogenesis of A. fumigatus infection remains insufficiently understood (6, 7).

The asexual reproduction of A. fumigatus leads to the formation and release of conidia into the atmosphere (8–10). Conidia are inhaled by humans and, due to their small size of 2 to 3 µm, easily reach the lung alveoli (reviewed in reference 6). Conidia are effectively cleared by the innate immune system and rarely pose a danger to healthy individuals (reviewed in references 7, 11, and 12); however, in patients with reduced mucociliary clearance or immune dysfunction, elimination of conidia can fail, leading to the development of IA.

Conidia are the first form of A. fumigatus to come in contact with lung epithelial and immune cells. Thus, the composition of the conidial surface has the potential to directly influence IA development. The surface of A. fumigatus conidia is composed of polysaccharides, proteins, dihydroxynaphthalene (DHN)-melanin pigment, and other secondary metabolites (13, 14). Although the polysaccharides that form the cell wall of A. fumigatus conidia have been extensively studied (7, 15–17), less is known about A. fumigatus conidial surface proteins with immune-modulatory functions. One well-studied protein is the RodA hydrophobin, which forms a cohesive, proteinaceous layer on resting conidia and masks Dectin-1-dependent host immune responses to increase fungal survival (18, 19). Despite this important function, a Δ*rodA* knockout retains full virulence in nonneutropenic mice immunosuppressed with cortisone acetate (20). We therefore reasoned that additional conidial surface proteins must protect conidia from recognition by the immune system. Using two complementary proteomics approaches, we identified the A. fumigatus cell wall proteome and characterized CcpA, a novel conidial cell-surface protein required for virulence, possibly via alteration of conidial innate immune recognition.

## RESULTS

### Conidial surface proteome analysis reveals the highly abundant CcpA protein.

To comprehensively elucidate the conidial surface proteome of A. fumigatus, we performed liquid chromatography-tandem mass spectrometry (LC-MS/MS) analysis of conidial cell wall proteins extracted by hydrogen-fluoride (HF)-pyridine treatment as well as of surface-exposed conidial peptides cleaved by trypsin. The HF-pyridine method was used to identify all proteins located in the cell wall, including glycosylphosphatidylinositol (GPI)-anchored proteins, whereas trypsin shaving was used to identify proteins that contain surface-exposed, trypsin-accessible regions. A total of 148 proteins were identified, including 116 proteins by HF-pyridine extraction and 48 proteins by trypsin shaving, with 15 common in both data sets (Table 1; see also Table S1 in the supplemental material). A total of 43 proteins contained a signal peptide for secretion (SignalP 4.1 Server [21]), 8 proteins contained predicted transmembrane helices (TMHMM Server v. 2.0 [22, 23]), and 8 proteins contained a GPI-anchored attachment signal (according to the data in reference 24). As expected, the RodA surface hydrophobin was found to be the most abundant protein by both approaches. Along with known cell wall proteins, we also elucidated multiple proteins with unknown functions. The most abundant of these uncharacterized proteins was the conserved hypothetical protein Afu1g13670 denoted CcpA (Table 1).

10.1128/mBio.01557-18.9 TABLE S1  Compilation of LC-MS/MS proteomics data collected in this study. Download TABLE S1, XLSX file, 0.4 MB. Copyright © 2018 Voltersen et al. 2018 Voltersen et al. This content is distributed under the terms of the Creative Commons Attribution 4.0 International license.

**TABLE 1:** LC-MS/MS analysis of highly abundant proteins extracted from resting conidia by HF-pyridine treatment (top 20)^a^

Protein name	Average no. of PSM/length		NSAF		Signal peptide	Brief description
	HF- pyridine	Trypsin shaving	HF- pyridine	Trypsin shaving		
RodA	0.49	0.58	0.13	0.23	+	Conidial hydrophobin
Grg1	0.25	0	0.06	0	−	Glucose repressible protein Grg1, putative
Afu1g13670 (CcpA)	0.24	0.16	0.06	0.06	+	Protein of unknown function; abundant in conidia
Afu3g11550	0.14	0	0.04	0	−	LEA domain protein
Afu6g12000	0.13	0	0.03	0	−	Uncharacterized protein
Afu3g11260	0.11	0	0.03	0	−	Ubiquitin (UbiC), putative
Scf1	0.10	0	0.03	0	−	Heat shock protein Awh11, putative
Afu6g10700	0.10	0	0.02	0	−	Chaperonin, putative
ConJ	0.08	0	0.02	0	−	Conidiation-specific protein (con-10), putative
Afu1g13780	0.07	0	0.02	0	−	Histone H4/histone H4.1
Htb1	0.07	0.06	0.02	0.02	−	Histone H2B
Afu2g11060	0.07	0	0.02	0	−	Acyl coenzyme A (CoA) binding protein family
Afu7g04030	0.07	0	0.02	0	−	Uncharacterized protein
Afu1g09890	0.07	0	0.02	0	−	Uncharacterized protein
Awh11	0.06	0	0.01	0	−	Chaperone/heat shock protein Hsp12, putative
Asp f 8	0.06	0	0.01	0	+	Allergen asp f 8
Afu3g03040	0.05	0	0.01	0	+	Uncharacterized protein
Afu2g13590	0.05	0	0.01	0	−	Uncharacterized protein/hypothetical protein
Afu2g11340	0.05	0	0.01	0	−	Phosphatidylglycerol/phosphatidylinositol transfer protein/ ML domain protein, putative
Afu4g02805	0.05	0	0.01	0	−	Asp hemolysin-like protein

a PSM, peptide spectrum matches; NSAF, normalized spectral abundance factor. NSAF data corresponding to the data set for each sample were calculated as follows: (number of PSM/length)/sum of all (PSM/length) values.

**FIG 1:**
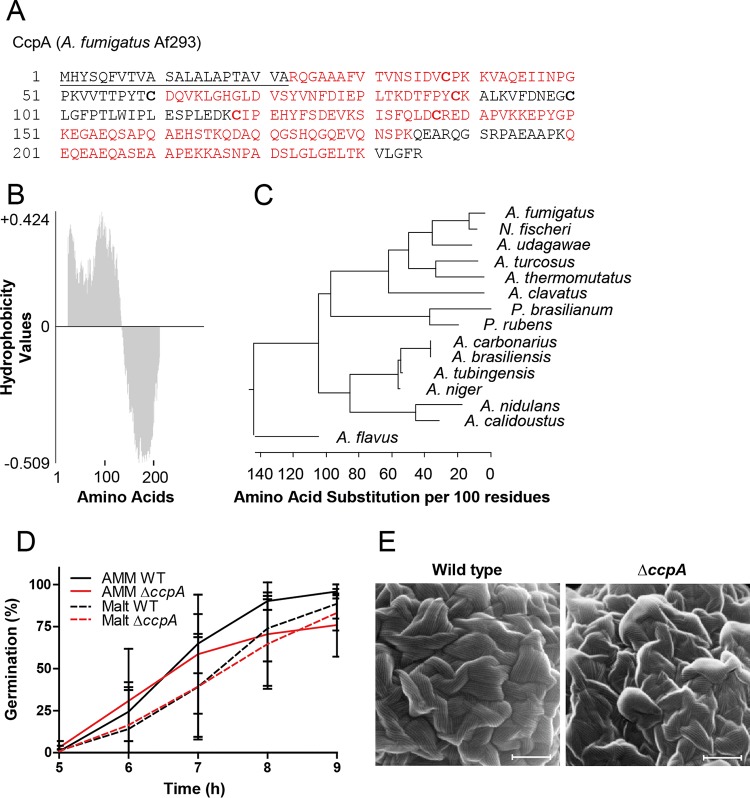
CcpA is a small amphiphilic protein found primarily in the genus *Aspergillus*. (A) FASTA sequence of A. fumigatus protein CcpA. The signal peptide for secretion is underlined. The six conserved cysteine residues are highlighted by bold characters. Peptides identified by LC-MS/MS are marked in red. (B) Hydrophobicity plot of the amino acid sequence of CcpA. The hydrophobicity values (*y* axis) are plotted against their corresponding amino acids (*x* axis) using the Kyte and Doolittle algorithm of VectorNTI software (Invitrogen). Hydrophobicity index values at pH 3.4 were determined by high-performance liquid chromatography (HPLC). (C) Phylogenetic analysis of CcpA proteins using maximum likelihood analysis. *P. brasilianum*, Penicillium brasilianum; *P. rubens*, Penicillium rubens. (D) Germination in RPMI media of wild-type (WT) and knockout conidia collected from AMM and malt agar plates. Data shown in panel D represent means ± standard deviations (SD) (*n* = 5). (E) Scanning electron microscopy (SEM) of resting conidia. Scale bars = 200 nm.

The levels of CcpA amino acid sequence coverage were 66.3% and 6.8% in the HF-pyridine and trypsin-shaving data sets, respectively (Fig. 1A). Only two peptides were identified by trypsin shaving, beginning at two consecutive lysine residues near the C-terminal end of the protein (KKASNPADSLGLGELTKVLGFR), suggesting that only a small portion of the protein is surface exposed. The *ccpA* gene is 757 bp in length, contains a single 48-bp intron at nucleotide 16, and encodes a predicted protein of 235 amino acids (deduced molecular mass, 25.7 kDa). CcpA contains a signal peptide for secretion and, interestingly, six conserved, invariant cysteine residues in the 15 organisms where it was readily identifiable (Fig. 1A; see also Fig. S1 in the supplemental material). The hydrophobicity plot of CcpA reveals an amphiphilic protein resembling RodA, with a hydrophobic N-terminal region and hydrophilic C terminus (Fig. 1B). CcpA is mainly found in *Aspergillus* species by BLAST analysis, and the closest homologues of A. fumigatus CcpA are in Neosartorya fischeri and A. udagawae (Fig. 1C).

10.1128/mBio.01557-18.1 FIG S1  Alignment of CcpA-containing fungi. ClustalW alignment was performed using DNASTAR MegAlign of the 15 sequenced fungal isolates where CcpA is readily identified. A consensus sequence is indicated for all residues that are found in all strains, including six invariant cysteines. Download FIG S1, PDF file, 0.2 MB. Copyright © 2018 Voltersen et al. 2018 Voltersen et al. This content is distributed under the terms of the Creative Commons Attribution 4.0 International license.

CcpA was deleted from wild-type A. fumigatus D141 (Δ*ccpA*) and correspondingly complemented (*ccpA*c) (Fig. S2). Deletion of *ccpA* did not affect radial growth on solid media (Fig. S3). We also observed no significant differences in the germination rates of wild-type and knockout conidia produced on *Aspergillus* minimal medium (AMM) or malt agar plates (Fig. 1D). Δ*ccpA* conidia showed no significant changes in comparison to wild-type conidia with respect to susceptibility to temperature stress (Fig. S3C), oxidative stress induced by H_2_O_2_ (Fig. S3D), or cell wall/membrane stressors (Fig. S4A). Finally, scanning electron microscopy (SEM) of the surface of resting Δ*ccpA* conidia revealed a rodlet layer identical to that of wild-type conidia, suggesting that deletion of *ccpA* does not significantly alter the surface characteristics of resting conidia. In addition, the surfaces of swollen conidia from the wild-type strain and from the Δ*ccpA* strain appeared similar (Fig. 1E; see also Fig. S4B).

10.1128/mBio.01557-18.2 FIG S2  Generation of A. fumigatus knockouts. (A) Schematic overview of the deletion strategy. The *ccpA* gene (AFUA_1G13670) was replaced by the deletion cassette, containing the *hph* hygromycin resistance gene, obtained from plasmid pΔ*ccpA* via homologous recombination using the 5′ and 3′ flanking regions. (B) Schematic overview of the complementation strategy. The complementation cassette was cut out of plasmid p*ccpA*c with HpaI and transformed into strain Δ*ccpA* to obtain locus-specific complementation. (C) Southern blot analysis of strains Δ*ccpA* and *ccpA*c. Confirmation of the deletion of *ccpA* and the complementation of the Δ*ccpA* strain was performed. Chromosomal DNA of parental wild-type strain D141 (lane 1), strain Δ*ccpA* (lane 2), and strain *ccpA*c (lane 3) was cut by ClaI. An 806-bp *ccpA* 5′ flanking PCR product was used as a probe. In the Δ*ccpA* strain, the band characteristic of the wild-type strain (4,425 bp) had disappeared. Instead, a band characteristic of a gene replacement at the locus (7,315 bp) was detected. The hybridization of the probe with the DNA of the complemented *ccpA*c strain resulted in a band with the size of 2,398 bp. Download FIG S2, PDF file, 0.3 MB. Copyright © 2018 Voltersen et al. 2018 Voltersen et al. This content is distributed under the terms of the Creative Commons Attribution 4.0 International license.

10.1128/mBio.01557-18.3 FIG S3  CcpA is not required for growth of A. fumigatus. (A) Growth on AMM (left) or on peptone (center) or malt agar (right) plates incubated for 3 days at 37°C. (B) Radial growth on different solid media. A total of 10^3^ conidia in a volume of 5 µl were centrally spotted on agar plates containing AMM, malt, or peptone. Agar plates were incubated for 96 h at 37°C. The radial growth was measured every 24 h. (C) The temperature sensitivity of conidia was measured by comparing survival rates after 1 h of incubation of 10^3^ conidia at −80°C, 22°C, 37°C, and 60°C to the CFU of the input. Results represent means ± SD (*n* = 3). (D) Sensitivity of conidia to oxidative stress. A total of 10^5^ conidia were incubated in the presence of 0, 0.2, 0.4, and 0.6 M H_2_O_2_. After 30 min of incubation, survival was determined via CFU counts. Results represent means ± SD (*n* = 3). Download FIG S3, PDF file, 0.6 MB. Copyright © 2018 Voltersen et al. 2018 Voltersen et al. This content is distributed under the terms of the Creative Commons Attribution 4.0 International license.

10.1128/mBio.01557-18.4 FIG S4  Cell wall stress responses do not require CcpA. (A) Representative images of droplet assays after serial dilutions of wild-type and Δ*ccpA* knockout conidia on standard cell wall-perturbing agents (Congo red and calcofluor white) and cell membrane-perturbing agents (SDS and terbinafine). Images were taken after 3 days of growth at 37°C. (B) The rodlet layer of resting conidia does not require CcpA. Representative images of wild-type and Δ*ccpA* knockout conidia grown on AMM and malt agar plates are presented. In all cases, germination was performed for 5 h in RPMI media. Each condition is represented by two magnifications. Download FIG S4, PDF file, 0.6 MB. Copyright © 2018 Voltersen et al. 2018 Voltersen et al. This content is distributed under the terms of the Creative Commons Attribution 4.0 International license.

### CcpA is produced in phialides and is detectable in the cell wall of resting conidia.

**FIG 2:**
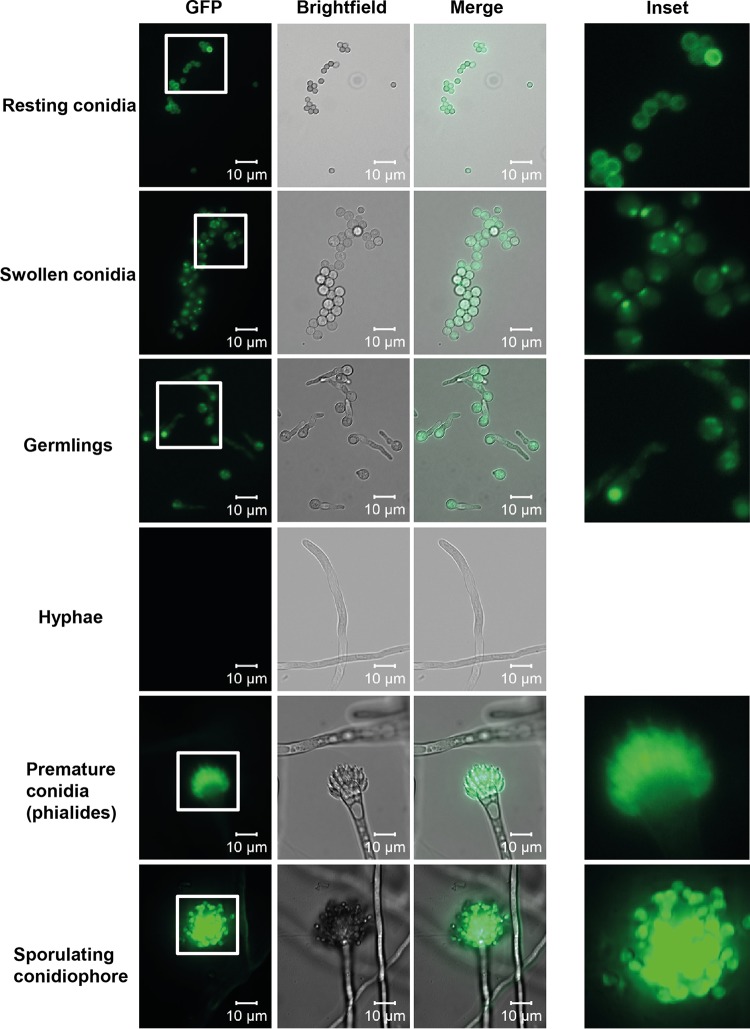
CcpA is localized to the cell wall. CcpA_eGFP was cultivated in AMM at 37°C for analysis of resting conidia (0 h), swollen conidia (4 h), germinating conidia (6 h), hyphae (10 h), and conidiophore formation (24 h). Samples were analyzed by light and fluorescence microscopy. Scale bars = 10 µm.

In order to confirm the localization of CcpA, a strain expressing CcpA fused to enhanced green fluorescent protein (eGFP) was generated (Fig. S5A and B). When grown in AMM at 37°C, no fluorescence was detectable in hyphae (Fig. 2). However, during conidiogenesis, a strong fluorescence signal deriving from the conidiophore was readily visible. The phialides also emitted the eGFP signal, which strongly indicated expression of CcpA in these conidium-forming cells. High-resolution imaging confirmed that resting conidia displayed signal near the surface (Fig. S5C and D). The eGFP fusion protein was also expressed in a Δ*ccpA* knockout strain to ensure that the localization was comparable to the expression of the fusion in wild-type resting conidia (Fig. S5E and F). During swelling of conidia, the eGFP signal appeared as scattered dots, which gradually accumulated in the vacuole, suggesting protein degradation (Fig. 2). In comparison, analysis of wild-type strain D141 as a control showed no fluorescent eGFP signal (Fig. S5G).

10.1128/mBio.01557-18.5 FIG S5  Generation of the recombinant strain CcpA_eGFP. (A) Plasmid pUC_GH_natp*ccpA_egfp.* (B) Southern blot analysis of wild-type and CcpA_eGFP strains. Ectopic integration of pUC_GH_natp*ccpA_egfp* in the genome of the wild-type strain was confirmed. Chromosomal DNA of parental strain D141 (lane 1) and the obtained transformants of CcpA_eGFP 1 to 10 (lanes 2 to 10) was cut by the use of BclI. An 894-bp *ccpA* PCR product was used as a probe. Beside the characteristic wild-type band (5.3 kb), one additional band characteristic of the ectopically integrated plasmid pUC_GH_natp*ccpA_egfp* was always detected in CcpA_eGFP strains 1 to 4 and 6 to 10. (C and D) Confocal laser scanning microscopy (C) and high-resolution Airyscan imaging (Zeiss) (D) of wild-type resting conidia expressing the CcpA_eGFP fusion construct. (E and F) High-resolution Airyscan imaging (Zeiss) (E) and 3D reconstruction (F) of Δ*ccpA* resting conidia expressing CcpA_eGFP. (G) The D141 wild-type strain was cultivated in AMM at 37°C for resting conidia (0 h), swollen conidia (4 h), germinating conidia (6 h), hyphae (10 h), and conidiophore formation (24 h). Samples were analyzed by light and fluorescence microscopy. Download FIG S5, PDF file, 0.3 MB. Copyright © 2018 Voltersen et al. 2018 Voltersen et al. This content is distributed under the terms of the Creative Commons Attribution 4.0 International license.

### CcpA is necessary for a normal surface proteome.

The surface localization and abundance of CcpA suggested a possible function in cell wall organization, and yet we observed no obvious cell surface differences in the knockout by SEM. Nor did we observe any alterations in the levels of mannose moieties of glycoproteins, chitin, or β-1,3-glucan in the cell wall of the knockout using concanavalin A-fluorescein isothiocyanate (concanavalin A-FITC), wheat germ agglutinin-FITC, or dectin-1 labeling, respectively (data not shown; the methods used were as described previously in reference 25). We also observed CcpA on the surface of both Δ*rodA* conidia lacking the rodlet layer and *pksP* mutant conidia lacking the melanin layer by trypsin shaving, suggesting that CcpA surface localization is likely not coupled to these conidial structures (M. G. Blango, T. Krüger, O. Kniemeyer, and A. A. Brakhage, unpublished data). To determine whether newly accessible surface proteins might be exposed on the Δ*ccpA* conidial surface, we performed cell surface trypsin shaving of resting and swollen conidia (5 h). LC-MS/MS analysis of resting conidia from both malt and AMM agar plates revealed an increase in the number of identified proteins unique to the Δ*ccpA* strain (Fig. S6A and B). Ten proteins were found exclusively on the surface of resting Δ*ccpA* conidia independently of the growth medium compared to the wild-type and complemented strains (Table S1). In swollen conidia, knockout spores grown on AMM agar displayed a similar phenotype, with a higher number of previously undetected proteins on the surface (Fig. S6C). Swollen conidia derived from malt agar plates were comparable to those derived from the wild-type and complemented strains, with only 17 proteins unique to the Δ*ccpA* knockout surface (Fig. S6D). The identification of newly exposed surface proteins of the Δ*ccpA* strain suggests that CcpA might limit the availability of *A. fumigatus* cell surface epitopes that would otherwise trigger inflammation upon each new encounter in healthy patients.

10.1128/mBio.01557-18.6 FIG S6  CcpA is required for a normal cell surface proteome. Venn diagrams show the overlap of proteins identified by LC-MS/MS after trypsin shaving of (A) resting conidia from AMM agar plates, (B) resting conidia from malt agar plates, (C) conidia from AMM agar plates swollen for 5 h in RPMI, and (D) conidia from malt agar plates swollen for 5 h in RPMI medium. Download FIG S6, PDF file, 0.3 MB. Copyright © 2018 Voltersen et al. 2018 Voltersen et al. This content is distributed under the terms of the Creative Commons Attribution 4.0 International license.

### CcpA reduces recognition by the innate immune system.

**FIG 3:**
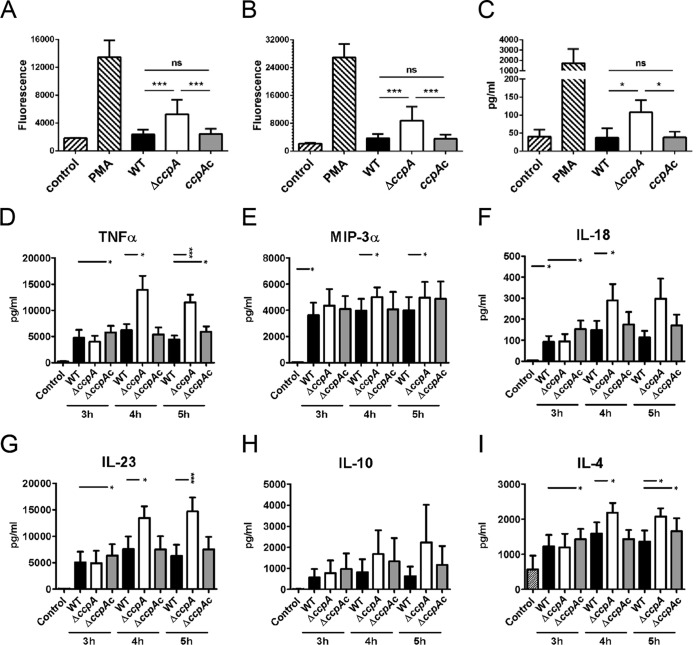
A. fumigatus conidia activate PMNs and moDCs. (A to C) Primary human PMNs were confronted with 3 h swollen conidia of the wild-type strain (black bars), the Δ*ccpA* strain (white bars), or the *ccpA*c strain (gray bars) at an MOI of 10 and analyzed for generation of intracellular ROS and IL-8 secretion. PMNs in media alone served as a negative control (control). PMNs treated with PMA served as a positive control. The bars show means ± SD of results from independent experiments performed with primary PMNs isolated from different donors. Neutrophil oxidative burst data are shown for (A) 60 min and (B) 120 min of cocultivation with swollen conidia as measured by the DCF-fluorescence intensity of ROS formation. Results represent means ± SD (*n* = 5). (C) Secretion of IL-8 was measured in supernatants after confrontation of PMNs with conidia for 3 h. Results represent means ± SD (*n* = 4). (D to I) MoDC cocultivation with A. fumigatus swollen conidia from the wild-type strain, Δ*ccpA* strain, or *ccpA*c strain was performed for 18 h. Prior to cocultivation, A. fumigatus conidia were swollen for 3, 4, or 5 h at 37°C. Supernatants were collected and analyzed with a multiplex immunoassay for (D) TNF-α, (E) MIP-3α, (F) IL-18, (G) IL-23, (H) IL-10, and (I) IL-4. Cytokine concentrations are shown in picograms per milliliter. Data represent means ± standard errors of the means of results from 4 independent experiments. Statistical significance was calculated by Student’s *t* test.

To test the hypothesis that CcpA contributes to innate immune evasion, we incubated primary polymorphonuclear leukocytes (PMNs), which mediate protection against invasive aspergillosis, with swollen conidia of the wild-type, Δ*ccpA*, and *ccpA*c strains. All three strains induced an oxidative burst in neutrophils over time (Fig. 3A and B). Formation of reactive oxygen intermediates (ROI) was strongly induced by Δ*ccpA* conidia after 60 min of coincubation compared with mock-infected PMNs (Fig. 3A). At this time point, ROI generation in response to wild-type and *ccpA*c conidia was detectable at only low levels and remained significantly lower than that seen in response to Δ*ccpA* conidia at 120 min (Fig. 3B). In line with this, PMNs confronted with Δ*ccpA* conidia secreted significantly more of the neutrophil chemoattractant interleukin-8 (IL-8) (Fig. 3C) (26), whereas PMNs incubated with either the wild-type or *ccpA*c strain released only basal levels of IL-8. These data suggest that CcpA limits recognition by PMNs.

We next analyzed swollen conidia of the Δ*ccpA* strain for the capacity to activate human monocyte-derived dendritic cells (moDCs). Formalin-fixed and swollen conidia (swollen for 3, 4, and 5 h) were coincubated with moDCs for 18 h. Interestingly, incubation of moDCs with Δ*ccpA* swollen conidia resulted in enhanced release of the proinflammatory cytokines tumor necrosis factor alpha (TNF-α), MIP-3a, IL-23, and IL-18 as well as the anti-inflammatory cytokines IL-10 and IL-4 (Fig. 3D to I). Taken together, these data suggest that CcpA on the surface of conidia limits recognition by both PMNs and DCs.

### CcpA contributes to both cell damage and virulence.

**FIG 4:**
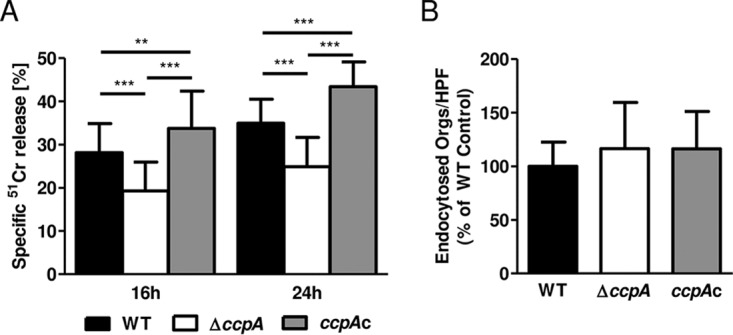
Deletion of *ccpA* limits induction of pulmonary epithelial cell damage. (A) The A549 pulmonary epithelial cell line was infected with the indicated strains of A. fumigatus for 16 and 24 h. The damage of epithelial cells was assessed by a ^51^Cr release assay. Results represent means ± SD (*n* = 3). (B) Endocytosis of swollen spores by A549 cells as determined by differential immunofluorescence analysis. Orgs, organisms; HPF, high-powered fields. Results represent means ± SD of results from three experiments performed in triplicate (>100 spores counted per replicate).

The capacity of A. fumigatus to damage pulmonary epithelial cells *in vitro* correlates with virulence in mouse models of IA (27–29). Therefore, we investigated the effects of the Δ*ccpA* deletion on the capacity of A. fumigatus to damage A549 pulmonary epithelial cells. Using a ^51^Cr release assay for cytotoxicity, the Δ*ccpA* strain was observed to cause significantly less damage to epithelial cells than the wild type after 16 and 24 h of incubation, suggesting decreased pathogenicity (Fig. 4A). This result was observed despite comparable levels of endocytosis for each strain (Fig. 4B).

The modified surface proteome, the enhanced capacity of the Δ*ccpA* knockout to stimulate PMNs and moDCs, and the reduced capacity to damage epithelial cells suggested that CcpA might be required for virulence. To test this assumption, we compared the virulence of the Δ*ccpA* strain with that of the wild-type and *ccpA*c strains in a nonneutropenic mouse model of IA. In this model, mice are immunosuppressed with cortisone acetate, and yet recruitment of neutrophils to the site of infection still occurs (30).

**FIG 5:**
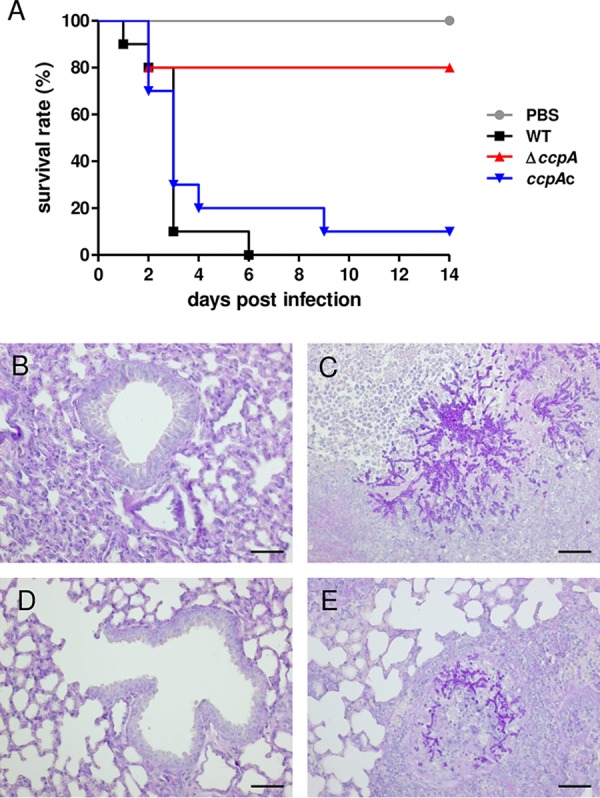
CcpA is required for virulence in a nonneutropenic mouse infection model. (A) Survival of nonneutropenic (cortisone acetate) mice after infection with wild-type, knockout, and complemented strains. Outbred CD-1 mice were intranasally infected with 1 × 10^6^ A. fumigatus conidia. Survival of infected mice was monitored for 14 days, and data are shown as a Kaplan-Meier plot (*n* = 10). (B to E) Histopathology from (B) PBS-treated mice (14 days postinfection [p.i.]), (C) WT-infected mice (3 days p.i.), (D) Δ*ccpA*-infected mice (14 days p.i.), and (E) *ccpA*c-infected mice (2 days p.i.). For histopathology, 4-µm sections of lungs were treated with periodic acid-Schiff stain (PAS; hyphae stained pink). Scale bars are 50 µm.

The virulence of the Δ*ccpA* strain was significantly attenuated (*P* < 0.002) in this mouse model (Fig. 5A). Over the course of infection, several mice infected with the Δ*ccpA* strain developed characteristic disease symptoms (signs of dyspnea, ruffled fur), but the majority of these animals recovered, which led to a final survival rate of 80% after 14 days. In contrast, by 6 days postinfection with wild-type conidia, all animals had succumbed to the infection. Infection with the complemented *ccpA*c strain resulted in 90% killing of animals. Histopathology revealed that the wild-type strain induced strong inflammation and extensive pulmonary fungal invasion, with destruction of bronchi and alveoli. In contrast, the lungs of mice infected with Δ*ccpA* conidia showed minimal evidence of inflammation and no fungi, suggesting early clearance of the conidia (Fig. 5B to E; see also Fig. S7).

10.1128/mBio.01557-18.7 FIG S7  Histopathology of cortisone acetate-treated, infected mice. (A) Additional images from cortisone acetate-treated mice infected with wild-type, knockout, or complemented conidia as shown in Fig. 5. Scale bars are 50 µm. (B) Higher (×630)-magnification histopathology images from PBS-treated, wild-type infected, or Δ*ccpA*-infected mice. For all histopathology, 4-µm sections of lungs were treated with periodic acid-Schiff stain (PAS; hyphae stained pink). Download FIG S7, PDF file, 0.4 MB. Copyright © 2018 Voltersen et al. 2018 Voltersen et al. This content is distributed under the terms of the Creative Commons Attribution 4.0 International license.

**FIG 6:**
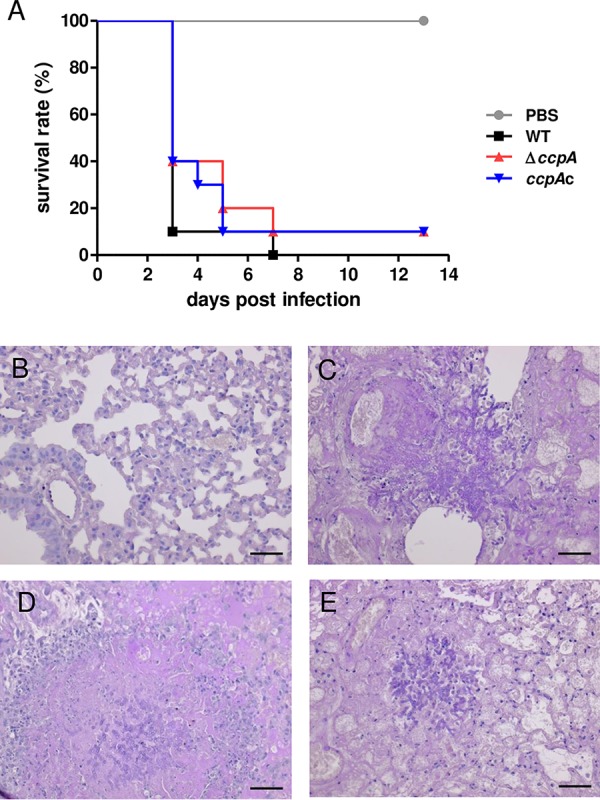
CcpA is dispensable for virulence in cyclophosphamide-treated mice. (A) Survival of neutropenic (cyclophosphamide) mice after infection with wild-type, knockout, and complemented strains. Outbred CD-1 mice were intranasally infected with 2 × 10^5^ A. fumigatus conidia. Survival of infected mice was monitored for 14 days; and data are shown as a Kaplan-Meier plot (*n* = 10). (B to E) Histopathology images from (B) PBS-treated mice (14 days p.i.), (C) WT-infected mice (3 days p.i.), (D) Δ*ccpA*-infected mice (5 days p.i.), and (E) *ccpA*c-infected mice (5 days p.i.). For histopathology, 4-µm sections of lungs were treated with periodic acid-Schiff stain (PAS; hyphae stained pink). Scale bars are 50 µm.

To demonstrate that the attenuated virulence of the Δ*ccpA* strain was due to enhanced clearance by immune effector cells, we tested virulence in neutropenic mice generated by cyclophosphamide treatment. In addition to neutropenia, these mice also exhibit increased lung inflammatory features and decreased regulatory T cell levels (31, 32). The Δ*ccpA* strain was as virulent as the wild-type strain in this mouse model (Fig. 6A). Histopathology indicated clear signs of invasive growth of hyphae in tissue for all strains (Fig. 6B to E). These results demonstrate that the Δ*ccpA* strain grows normally in the mouse lung and that the drastically attenuated virulence of this strain in nonneutropenic mice is likely due to interaction with the innate immune system.

### The CcpA protein elicits a normal immunogenic T cell response.

To test whether CcpA might play an active role in suppression of the adaptive immune response, we studied the reactivity of moDCs and recall T cell responses of healthy human donors against a recombinant CcpA protein purified as described in Materials and Methods (Fig. S8A). Following *in vitro* stimulation with recombinant CcpA, moDCs upregulated various costimulatory molecules (CD80, CD83, CD86, and CD40) and antigen-presenting molecules (HLA-ABC and HLD-DR) as well as proinflammatory cytokines compared to unstimulated controls (Fig. S8B and C), indicating that recombinant CcpA protein has a boosting effect on the immune system via activated DCs.

10.1128/mBio.01557-18.8 FIG S8  Recombinant CcpA elicits a heightened cytokine response from moDCs. (A) Coomassie-stained SDS-PAGE gel of purified His_6_-CcpA_23 to 218_. Lane 1, cell lysate containing 8 M urea; lane 2, eluted His_6_-CcpA_23 to 218_ protein after co-chelate chromatography; lane 3, pure His_6_-CcpA_23 to 218_ after buffer exchange. (B) Coincubation of 1 µg/ml recombinant CcpA protein with moDCs for 18 h. Stimulation with 1 µg/ml LPS served as a positive control. Data representing maturation of moDCs are displayed as mean fluorescence intensity (MFI) values measured by flow cytometry. (C) Secretion of cytokines upon stimulation with recombinant CcpA. Cytokine release of moDCs stimulated with 5 µg/ml CcpA protein was analyzed by multiplex immunoassay using culture supernatants. Cytokine concentrations are shown in picograms per milliliter. Data represent means + standard errors of the means of results from *n* = 3 (A) and *n* = 3 (B) independent experiments. Statistical significance was calculated by Student’s *t* test. Download FIG S8, PDF file, 0.2 MB. Copyright © 2018 Voltersen et al. 2018 Voltersen et al. This content is distributed under the terms of the Creative Commons Attribution 4.0 International license.

**FIG 7:**
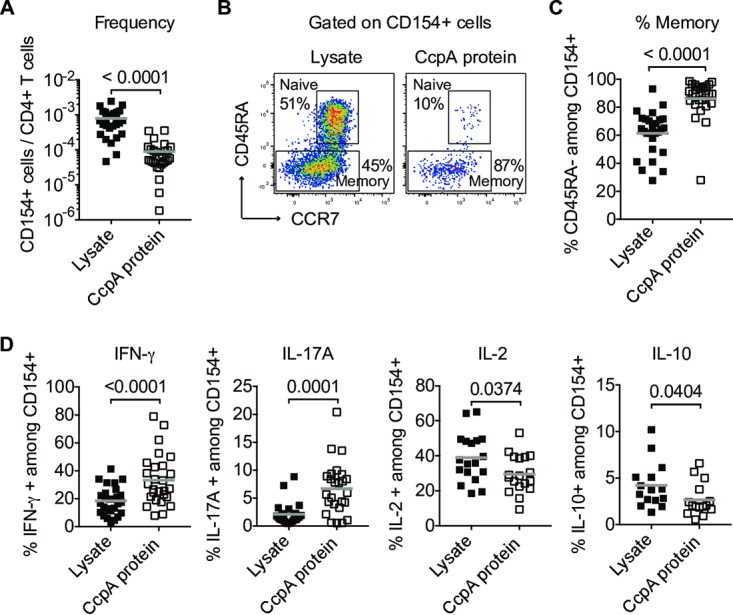
The recombinant CcpA protein is an immunogenic T cell target in healthy humans. (A) Frequencies of reactive CD154^+^ Tcons among CD4^+^ T cells (*n* = 27, from seven independent experiments). (B) Enriched CD154^+^ cells were stained for phenotypic markers to discriminate between naive (CD45RA^+^ CCR7^+^) and memory (CD45RA^−^) cells. (C) Statistical analysis of the memory phenotype of crude cell extract or recombinant CcpA protein-reactive CD154^+^ cells (*n* = 27). (D) Magnetically preenriched CD154^+^ cells were stained for cytokine expression. Percentages of cytokine-expressing cells among CD154^+^ T cells are given. IFN-γ and IL-17A, *n* = 27; IL-2, *n* = 18; IL-10, *n* = 15; data represent results from seven independent experiments. Each symbol in panels A, C, and D represents one donor. Horizontal bars indicate mean values. Statistical differences were determined by two-tailed paired Wilcoxon test.

To confirm this finding, we tested whether CcpA induces a specific immune response during natural exposure. Humans are continuously exposed to A. fumigatus; thus, *in vivo* primed T cells specific for immunogenic A. fumigatus proteins are readily detectable in the blood (33, 34). Enrichment of antigen-reactive CD154^+^ T cells (Tcons) following *ex vivo* CcpA stimulation of human blood cells allowed identification of *in vivo* primed CcpA-specific T cells according to cell surface phenotype and effector cytokine expression results. Specific T cells against CcpA could be clearly detected in healthy donors, albeit at a lower frequency than that seen following stimulation with whole-conidium extracts (Fig. 7A). However, compared to total A. fumigatus-specific T cells, the CcpA-specific T cells showed increased frequencies of memory cells (CD45RA^−^) (Fig. 7B and C) and inflammatory effector cytokine-expressing cells (Fig. 7D) as follows: for IFN-γ, a mean of 35.1% and a range of 7.9% to 78.9%; for IL-17A, a mean of 6.2% and a range of 0.53% to 20.4%). However, the level of expression of the anti-inflammatory cytokine IL-10 was reduced (mean, 2.68%; range, 0.55% to 6.59%) (Fig. 7D). These data identify an *in vivo* primed phenotype of CcpA-specific human T cells, suggesting that within the A. fumigatus proteome CcpA belongs to the group of immunogenic proteins (34). Overall, these data indicate that the CcpA protein has no protein-intrinsic immunosuppressive activity but rather seems to exert its function by limiting the recognition of resting conidia by altering the accessibility of the conidial surface, which, in the context of the immunosuppressed host, serves as a growth advantage for the fungus.

## DISCUSSION

Proteins on the surface of conidia are likely to interact with the human immune system upon inhalation. In recent years, several studies have enlarged the list of A. fumigatus conidial surface proteins. Asif et al. treated intact resting A. fumigatus conidia with the cell wall-lyzing enzyme β-(1,3)-glucanase and identified 25 different proteins (35). Similarly, Suh et al. found 52 proteins enriched in cell wall fractions by alkaline treatment of resting conidia (36). Here, we provide the most extensive overview to date of the conidial surface proteome, with 116 proteins identified by HF-pyridine extraction and 47 proteins identified by trypsin shaving, for a total of 148 unique cell wall proteins. We detected 22/25 of the proteins found by Asif et al. and 33/52 of the proteins found by Suh et al. in the cell wall of resting conidia. By comparing proteins identified by two complementary techniques, we were able to assess several aspects of the cell wall proteome. The HF-pyridine extraction technique releases cell wall and GPI-anchored proteins and uncovers the most abundant proteins in the cell wall, whereas the trypsin-shaving approach assesses the surface-exposed fraction of proteins that are trypsin accessible. Across all strains and conditions tested in the manuscript, including Δc*cpA* knockout conidia, we observed 701 unique proteins associated with the cell wall, including many known cell wall-associated proteins but also a number of unknown proteins (see Table S1 in the supplemental material) (35, 36).

A large fraction of the identified proteins had no assigned biological function. The most abundant of these proteins was CcpA. Although this protein has a signal peptide, its amino acid sequence contains no other domain suggestive of cell wall localization. However, it was previously reported that CcpA is part of the conidial surface proteome (35). Localization studies performed with an eGFP-fusion protein revealed that CcpA was produced only in specific cell types, in the phialide layer formed on the premature conidiophore vesicle, as well as along the surface of conidiophores and resting conidia, consistent with the finding that CcpA is a resting-conidium-enriched protein (36). These findings are consistent with recent observations that *ccpA* appears to be regulated by the three transcription factors central to asexual development, BrlA, WetA, and AbaA (37), and that it contains a conserved, canonical AbaA binding site, with motif “CATTCC” (MEME analysis; reference 38 and data not shown). Due to the high abundance of CcpA in the cell wall and the cysteine-rich amino acid sequence, one may speculate that CcpA acts as a scaffold for the conidial hydrophobin layer. However, SEM pictures revealed that the Δ*ccpA* knockout displays organized and compact rodlet structures over the entire resting conidial surface, similarly to wild-type conidia. Nevertheless, its amphiphilic character supports the idea of self-assembly through aggregation and a potential structural role in the organization of the conidial cell wall.

Intriguingly, proteomics analysis revealed new trypsin-accessible, surface-exposed proteins on Δ*ccpA* resting conidia that could potentially contribute to its enhanced recognition and/or clearance. These phenotypes were also observed on the surface of swollen conidia from AMM agar plates, but not malt agar plates, perhaps suggesting a role for CcpA in modulating the surface environment under conditions of nutrient limitation or stress. The altered protein composition of the conidial surface offers compelling evidence for a role of CcpA in masking conidial surface antigens that could otherwise trigger an immune response in the host. It is not clear if these newly exposed proteins are actively hidden or if they just inherit the space made available by a missing CcpA protein. Although the presented evidence strongly suggests that CcpA limits immune recognition by PMNs and DCs, it is also formally possible that CcpA contributes indirectly to these fungal phenotypes.

The abundant surface localization and altered surface proteome of the CcpA knockout led us to test the contribution of CcpA to virulence. The Δ*ccpA* knockout stimulated greater proinflammatory cytokine secretion and reactive oxygen species (ROS) production by PMNs and cell surface marker expression by moDCs. In nonneutropenic mice immunosuppressed with cortisone acetate, the Δ*ccpA* strain showed significantly attenuated virulence. Interestingly, the knockout strain exhibited virulence comparable to that of the wild-type strain in cyclophosphamide-treated mice, with the mice succumbing to invasive fungal growth (30, 39). In addition to neutropenia, these mice also exhibited other alterations to the normal immune response, including increased infiltration and abundance of lung eosinophils, decreased regulatory T cell levels, and loss of some subsets of fast-growing epithelial cells, among others (31, 32, 40). In addition to showing the importance of CcpA in promoting maximal virulence, this experiment also demonstrated that the Δ*ccpA* deletion strain grows normally in an immunocompromised host, suggesting that reduced virulence in nonneutropenic mice was not solely due to a generalized growth defect.

In cell culture experiments, recombinant CcpA does not appear to be toxic to host cells (data not shown), consistent with our experiments in human T cells. In particular, our analysis of the *in vivo* immunogenicity of the purified CcpA protein suggests that the protein triggers a normal adaptive immune response upon natural exposure in healthy humans. We see no evidence for a protein-intrinsic immunosuppressive function or immunologic inertness. Thus, it seems that the presence of the protein on resting conidia is important for a normal surface structure. In the absence of CcpA, other proteins become more accessible on the conidial surface, leading to increased immune recognition and activation, in particular, by neutrophilic granulocytes. Although it requires further study, we hypothesize that CcpA serves as a major structural and stealth protein on the A. fumigatus conidial surface to prevent immune recognition by the innate immune system of the host.

## MATERIALS AND METHODS

### Strains and cultivation conditions.

All strains used in this study are listed in Table S2 in the supplemental material. The clinical isolate D141 (41) served as the wild-type progenitor for all A. fumigatus strains constructed. Unless otherwise stated, A. fumigatus was cultivated at 37°C on *Aspergillus* minimal medium (AMM) agar plates or in AMM containing 50 mM glucose and 70 mM NaNO_3_ as previously described (42, 43). Media were supplemented with 0.1 µg/ml pyrithiamine (Sigma-Aldrich) or 150 µg/ml hygromycin (InvivoGen) when required. Conidia were harvested in sterile water. Escherichia coli strains were cultivated on LB agar plates or in LB supplemented with 50 µg/ml kanamycin sulfate (AppliChem) at 37°C.

10.1128/mBio.01557-18.10 TABLE S2  Strains, plasmids, and oligonucleotides used in this study. Download TABLE S2, PDF file, 0.3 MB. Copyright © 2018 Voltersen et al. 2018 Voltersen et al. This content is distributed under the terms of the Creative Commons Attribution 4.0 International license.

### HF-pyridine extraction of conidial surface proteins.

Proteins were extracted as previously described (44). A. fumigatus ATCC 46645 conidia grown for 7 days on malt agar were harvested, washed with sterile water, frozen in liquid nitrogen, and lyophilized. Lyophilized conidia (100 mg) were treated with 750 µl hydrogen-fluoride (HF)–pyridine (Sigma-Aldrich) and neutralized with 1 M ice-cold Tris base, and proteins were precipitated with trichloroacetic acid (TCA)-acetone. After centrifugation for 20 min at 1,700 × *g* at 4°C, the pellet was rinsed twice in ice-cold acetone–1 mg/ml dithiothreitol (DTT) before drying was performed for 15 min at room temperature. The extracted proteins were resuspended in 4 M urea–100 mM ammonium bicarbonate and sonicated for 20 min. After centrifugation at 20,800 × *g* for 20 min at 16°C, protein concentration was determined using the Bio-Rad protein assay (Bio-Rad) (45).

### Trypsin shaving of surface-exposed peptides.

Seven-day-old resting and swollen conidia (5 h in RPMI 1640) from malt agar plates were washed twice with 25 mM ammonium bicarbonate and collected by centrifugation (1,800 × *g* for 10 min). Samples were treated with 5 µg trypsin (Serva) for 5 min at 37°C. Spores were separated from cleaved peptides using a 0.2 µm-pore-size cellulose acetate filter (Sartorius) followed by inhibition of trypsin with formic acid (Sigma-Aldrich). Peptides were dried using a SpeedVac concentrator (Thermo-Fisher), resuspended in 25 µl of 2% (vol/vol) acetonitrile (ACN)–0.05% (vol/vol) trifluoroacetic acid, and centrifuged for 15 min through a 10-kDa-cutoff microcentrifuge column (VWR). Due to continuous optimization of our sample preparation, the samples for swollen conidia only were instead passed through a 0.22-µm-pore-size Spin-X cellulose acetate spin filter (Corning Costar), and the trypsin peptides were removed *in silico*.

### LC-MS/MS analysis of HF-pyridine-extracted peptides.

The in-solution digestion and subsequent LC-MS/MS analysis were carried out by Toplab GmbH as described previously (46) with some modifications. Briefly, one-dimensional (1D) nano-LC separation was performed with a multidimensional liquid chromatography system (Ettan MDLC; GE Healthcare). Peptides were loaded on an RPC trap column (LC Packings) with a flow rate of 6 µl per min (loading buffer, 0.1% [vol/vol] formic acid; trap column, C_18_ PepMap 100; 5-µm bead size; 300-µm inner diameter [i.d.]; 5-mm length) and subsequently separated by an analytical column (LC Packings) (C_18_ PepMap 100; 3 µm bead size; 75-µm i.d.; 15-cm length) with a 120-min gradient (buffer A, 0.1% [vol/vol] formic acid; buffer B, 84% [vol/vol] acetonitrile and 0.1% [vol/vol] formic acid) at a flow rate of 260 nl/min. The gradient was 0 to 30% buffer B for 80 min, 30% to 60% buffer B for 30 min, and 100% for 10 min.

An LTQ Orbitrap XL mass spectrometer (Thermo Fisher Scientific Company) coupled online with the nanoLC system was used. For electrospray ionization, a distal coated SilicaTip (FS-360-50-15-D-20) and a needle voltage of 1.4 kV were used. The MS method consisted of a cycle combining one full MS Orbitrap scan (resolution, 60,000; mass range, 300 to 2,000 *m*/*z*) with five data-dependent LTQ MS/MS scans (acquisition parallel to Orbitrap scans; 35% collision energy). The dynamic exclusion was set to 30 s.

### Database search and data analysis of HF-pyridine-extracted peptides.

MS/MS data were searched against the UniProt protein database using Mascot version 2.1.04 (Matrix Science). Search parameter settings were the following: (i) enzyme, trypsin; (ii) fixed modifications, carbamidomethyl (C); (iii) variable modifications, oxidation (M); (iv) peptide mass tolerance, 2 Da; (v) MS/MS tolerance, 0.8 Da; (vi) peptide charge, 1+, 2+, and 3+; (vii) instrument, electrospray ionization trap (ESI-TRAP); (viii) maximum number of missed cleavages allowed, 1.

Database search results were further processed with Scaffold version 3.0 (Proteome software) using the following parameters: minimum number of unique peptides = 2, minimum peptide identification probability = 95% (47). Normalized spectral counts were determined to assess relative protein abundance levels. Proteins were considered when at least two spectral counts were measured in two of three biological replicates.

### LC-MS/MS analysis of trypsin-shaved surface peptides.

Proteomics analysis was performed on an Ultimate 3000 RSLC nanoLC instrument coupled to a QExactive HF mass spectrometer (Thermo Fisher Scientific Company). Tryptic peptides were trapped for 4 min on an Acclaim PepMap 100 column (2 cm by 75 µm, 3-µm pore size) at a flow rate of 5 µl/min. Subsequently, the peptides were separated on an Acclaim PepMap column (50 cm by 75 µm, 2-µm pore size) using a binary gradient (buffer A, 0.1% [vol/vol] formic acid–H_2_O; buffer B, 0.1% [vol/vol] formic acid–90:10 [vol/vol] ACN/H_2_O) as follows: 0 to 4 min at 4% buffer B, 5 min at 8% buffer B, 20 min at 12% buffer B, 30 min at 18% buffer B, 40 min at 25% buffer B, 50 min at 35% buffer B, 57 min at 50% buffer B, 62 to 65 min at 96% buffer B, and 65.1 to 90 min at 4% buffer B. Positively charged ions were generated by the use of a nanospray Flex ion source (Thermo Fisher Scientific Company) and a stainless steel emitter with a spray voltage of 2.2 kV. Ions were measured in full MS/data-dependent (dd) MS2 (Top10) mode; i.e., precursors were scanned at *m/z* 300 to 1,500 (R, 60,000 full width at half maximum [FWHM]; AGC target, 1e6; maximum injection time [IT], 100 ms). Fragment ions generated in the higher-energy collisional dissociation (HCD) cell at 30% normalized collision energy were scanned using N_2_ (R, 15,000 FWHM; AGC target, 2e5; maximum IT, 100 ms) in a data-dependent manner (dynamic exclusion, 30 s).

### Database search and data analysis of trypsin-shaved surface peptides.

MS/MS data were searched against the A. fumigatus Af293 database of the Aspergillus Genome Database (AspGD) using Proteome discoverer 1.4 and the algorithms of Mascot 2.4.1, Sequest HT, and MS Amanda. Two missed cleavages were allowed for tryptic peptides, the precursor mass tolerance was set to 10 ppm, and the fragment mass tolerance was set to 0.02 Da. The dynamic modification was oxidation of Met. At least 2 peptides per protein and a strict target false-discovery rate (FDR) of <1% were required for positive protein hits.

### Manipulation of DNA, Southern blotting.

Manipulation of DNA was carried out according to standard procedures (48). Sequence information was obtained from the Aspergillus Genome Database (AspGD; http://www.aspergillusgenome.org) (49, 50). Chromosomal DNA of A. fumigatus was isolated using a MasterPure Yeast DNA purification kit (Epicentre Biotechnologies). For Southern blot analysis, chromosomal DNA of A. fumigatus was digested with the indicated restriction enzymes (New England Biolabs). DNA fragments were separated in an agarose gel and blotted onto Hybond N+ nylon membranes (GE Healthcare Bio-Sciences). Labeling of DNA probes, hybridization, and detection of DNA-DNA hybrids were performed using digoxigenin (DIG) labeling mix, DIG Easy Hyb, and a CDP-Star ready-to-use kit (Roche Applied Science), respectively, according to the manufacturer’s recommendations.

### Genetic manipulation of A. fumigatus.

Plasmids are listed in Table S2 and oligonucleotide sequences in Table S2. Plasmid pΔ*ccpA* was generated using plasmid pSK397 (51, 52). pΔ*ccpA* contained the hygromycin B phosphotransferase gene (*hph*) under the control of the *gpdA* promoter and the *trpC* terminator of Aspergillus nidulans (see Fig. S1A in the supplemental material). The *ccpA* locus of A. fumigatus was targeted with a construct containing 1.2-kb flanking regions (5′-*ccpA* and 3′-*ccpA*). Oligonucleotides *ccpA*-1 to *ccpA*-8 encoded restriction sites at the 5′ end for subsequent cloning steps and were used to amplify 5′-*ccpA* and 3′-*ccpA* by nested PCR. 5′-*ccpA* and 3′-*ccpA* were digested with NotI/XmaI and EcoRI/PacI, respectively, and were ligated end to end into pBluescript II S.K.(+)_*Pac*I (53). The hygromycin-resistance cassette was excised with SfiI from pSK397 and cloned into pBluescript to yield pΔ*ccpA*.

To complement strain Δ*ccpA*, plasmid p*ccpA*c was designed. *ccpA* and its 5′ and 3′ flanking regions were PCR amplified using primers v831/sv832_*ptrA*_re and sv833_*ptrA*_fw/sv834. The pyrithiamine resistance cassette was amplified from pSK275 with primers sv197/sv198. p*ccpA*c was generated using a GeneArt seamless cloning and assembly kit (Invitrogen) according to the manufacturer's instructions (Fig. S1B).

For localization studies, plasmid pUC_GH_natp*ccpA*_*egfp* was generated. Primers natp*ccpA*_fw_*Acc*65I and *ccpA*_re_*Xma*I encoded additional 5′ restriction sites and were used to amplify *ccpA* and an upstream 1,602-bp fragment comprising the native promoter. The amplified natp*ccpA* DNA fragment was ligated in frame with *egfp* into pUC_GH to yield plasmid pUC_GH_natp*ccpA*_*egfp*. Prior to transformation, the insertions of plasmids pΔ*ccpA* and p*ccpA*c were excised by NotI/PacI and HpaI, respectively. Knockouts of A. fumigatus were generated by homologous recombination following the transformation of protoplasts (54). pUC_GH_natp*ccpA*_*egfp* was incorporated into the genome of A. fumigatus D141 through ectopic integration. To study the functionality of the CcpA-eGFP fusion protein, the Δ*ccpA* mutant was complemented with a *ccpA*-*egfp* fusion construct. For this purpose, plasmid pSK275_natp*ccpA*_*egfp* was generated. The corresponding oligonucleotides nosT_rev_KpnI and ccpAp_for_KpnI were used with Flash-Phusion master mix (Thermo Scientific) to amplify the *ccpAp-ccpA-egfp* cassette from plasmid pUC_GH_natp*ccpA*_*egfp*. The 3.4-kb PCR product was subcloned into pJet2.1 following the protocol of a CloneJET PCR cloning kit (Thermo Scientific). Then, the construct was inserted as a KpnI fragment into plasmid pSK275. Plasmid pSK275_natp*ccpA*_*egfp* was used to transform protoplasts of the Δ*ccpA* mutant. Pyrithiamine-resistant transformants were screened for fluorescence, and the complete *ccpA-egfp* sequence was verified by PCR with oligonucleotides nosT_rev and ccpAp_for.

### Production of recombinant CcpA protein in E. coli.

A truncated version of CcpA lacking the 22-amino-acid secretion signal and 17 amino acids of the C terminus was produced. For this purpose, a synthetic gene with optimized codon usage for E. coli (Invitrogen, Life Technologies) was designed. By using oligonucleotides *ccpA*_23*Bam*HIfw/*ccpA*_218*Hin*dIIIre, *ccpA*_23-218 was amplified from pMAT_*ccpA*_23-218. The *ccpA*_23-218 amplified PCR product was cloned into the BamHI/HindIII sites of plasmid pET28aH6TEV to give plasmid pET28aHT*ccpA*_23-218, which was used to transform E. coli strain BL21(DE3). His_6_-*ccpA*-23-218 was produced by autoinduction in E. coli BL21(DE3) cells grown at 25°C in 1 liter of ZYP-5052 containing 0.5% (vol/vol) glycerol, 0.05% (wt/vol) glucose, 0.2% (wt/vol) α-lactose, and appropriate supplements (55). Cells (10 g by wet weight) were collected by centrifugation, resuspended in 100 ml lysis buffer, and homogenized using an Emulsiflex C5 high-pressure homogenizer (Avestin). Urea (8 M) was directly added to the lysate and dissolved. The fusion protein was isolated from clarified supernatant using a 5 ml HiTrap Talon crude column (GE Healthcare). Desalting and buffer exchange of His_6_–Afu1g13670_23-218 were performed using a 53-ml HiPrep 26/10 desalting column (GE Healthcare) by elution with 50 mM HEPES (pH 8.0)–300 mM NaCl.

### Growth assays.

Growth tests were performed on malt agar and on AMM agar plates containing either 50 mM glucose or 1% (wt/vol) peptone as the sole carbon source. Conidia (1 × 10^3^ in 5 µl) were centrally spotted on agar plates in three technical replicates. The plates were incubated for 96 h, and radial growth was measured every 24 h.

The rate of germination was determined in RPMI media for spores collected from AMM or malt agar plates incubated at 37°C for 5 days. At 5, 6, 7, 8, and 9 h after the initiation of germination, an aliquot of spores was counted to determine the ratio of spores undergoing germination.

To analyze the susceptibility of conidia to oxidative stress, 1 × 10^5^ spores were treated with 0, 0.2, 0.4, or 0.6 M H_2_O_2_ in a total volume of 1 ml for 30 min at room temperature. For testing the susceptibility of conidia at different temperatures, 1 × 10^5^ spores were incubated at −80°C, 22°C, 37°C, or 60°C for 1 h. After treatment, spore suspensions were diluted in water containing 0.001% (vol/vol) Tween 80 to a final concentration of 1 × 10^3^/ml. A 100-µl volume of each sample was plated on Sabouraud agar. After 24 h at 37°C, CFUs were counted and compared to an input that was plated from the initial dilution as described above.

### Microscopy.

For fluorescence and light microscopic analysis, 50 µl AMM on glass coverslips in a wet chamber was inoculated with 2 × 10^4^ conidia. Samples were analyzed after 0, 4, 6, 10, and 24 h using a Zeiss Axio Imager.M2 (Zeiss). Images were taken with an AxioCam MRm and analyzed by the use of AxioVision SE64 Rel. 4.9.1 imaging software (Zeiss). For confocal scanning laser microscopy, a Zeiss LSM 780 instrument was used along with an Airyscan detector where noted. Images were processed using the ZEN software package from Zeiss.

For scanning electron microscopy (SEM) analysis, resting conidia from mycelia grown on AMM agar plates for 5 days were collected using an electrically conductive and adhesive tag (Leit-Tab; Plano GmbH). Samples for resting conidia were ﬁxed for 24 h in a desiccator containing a solution of 25% (vol/vol) glutaraldehyde, whereas swollen conidia were fixed in 2.5% (vol/vol) glutaraldehyde. Further preparation and scanning electron microscopy were carried out as previously described (56).

### Interaction of A. fumigatus with PMNs.

Human PMNs were purified from venous blood of healthy volunteers by density gradient centrifugation using Polymorphprep (Progen Biotechnik GmbH) as described previously (57). Immune cell fractions were resuspended in RPMI 1640 (Gibco) containing 5% (vol/vol) heat-inactivated fetal calf serum (FCS) (Biochrome). Conidia were harvested after 5 days on malt agar plates. Swelling was induced by incubating conidia in RPMI 1640 with 5% (vol/vol) heat-inactivated FCS for 3 h at 37°C. Oxidative burst was determined by the 2′,7′-dichlorofluorescein (DCF) assay (58). PMNs were preincubated with 1 µM 2′,7′-dichlorofluorescin diacetate (DCFH-DA) (Sigma-Aldrich) at room temperature for 30 min. In a 96-well ﬂat-bottom black optical plate (Greiner), 50 µl of 4 × 10^6^ PMNs/ml were confronted with 50 µl of 4 × 10^7^ swollen conidia/ml (multiplicity of infection [MOI] = 10) in RPMI 1640 with 5% (vol/vol) heat-inactivated FCS. PMNs in media alone served as a negative control, whereas cells treated with 50 ng/ml phorbol-12-myristate-13-acetate (PMA; Sigma-Aldrich) served as a positive control. Fluorescence was measured using a Tecan Inﬁnite 200 plate reader at 37°C with excitation at 485 nm and emission at 535 nm over a period of 3 h. Release of IL-8 by PMNs was measured in supernatants from samples after 3 h of confrontation using Luminex technology (Procarta immunoassay kit—Magnetic Beads, Human) according to the manufacturer's instructions.

### Interaction of A. fumigatus and recombinant CcpA with human moDCs.

Human moDCs were generated from monocytes, which were isolated from peripheral blood mononuclear cells using CD14^+^ microbeads (Miltenyi Biotec) as described previously (59). Purity of moDCs was determined by flow cytometry (FACSCalibur; BD Biosciences) as >85% CD1a positive (anti-CD1a-allophycocyanin [anti-CD1a-APC]; BD Biosciences) and >95% CD14 negative (anti-CD14-FITC; BD Biosciences).

For interaction studies, conidia were harvested after 5 days of growth on malt agar plates. Swelling was induced by incubating conidia in RPMI medium (Lonza) for 3, 4, or 5 h at 37°C. Fixation was achieved using 3% (vol/vol) formaldehyde for 1 h. Fixed conidia were subsequently pelleted by centrifugation. Pellets were washed with sterile water and RPMI medium containing 10% (vol/vol) heat-inactivated fetal calf serum (FCS) prior to stimulation.

For interaction studies, 6 × 10^5^ moDCs were stimulated for 18 h with 6 × 10^5^ swollen conidia (MOI of 1) in 300 µl RPMI medium containing 10% (vol/vol) FCS. Maturation was analyzed by flow cytometry (anti-CD80-APC, anti-CD83-phycoerithrin [anti-CD83-PE], anti-CD86-FITC, anti-HLA-DR–PE; BD Biosciences). Cytokine release from culture supernatants was analyzed by TNF-α and IL-10 enzyme-linked immunosorbent assays (ELISA) (TNF-α ELISA, MAX Standard Sets, BioLegend; IL-10 ELISA, R&D Systems).

Stimulation with recombinant protein was performed for 18 h with 6 × 10^6^ moDCs in 300 µl RPMI medium containing 10% (vol/vol) FCS and 1 µg/ml of endotoxin-free purified CcpA (Pierce high-capacity endotoxin removal resin; Thermo Fisher Scientific) (0.009 endotoxin units [EU]/µg) or 1 µg/ml lipopolysaccharide (LPS). Then, maturation of moDCs was analyzed by flow cytometry as described above (and, in addition, with anti-HLA-ABC [Becton, Dickinson] and anti-CD40-FITC and anti-CCR7-APC [Miltenyi Biotec]).

### Pulmonary epithelial cell damage assay.

The A549 type II pneumocyte cell line was cultivated in F-12 K medium (American Type Culture Collection) containing 10% (vol/vol) fetal bovine serum (FBS) (Gemini Bio-Products), streptomycin, and penicillin (Irvine Scientiﬁc) in 5% (vol/vol) CO_2_ at 37°C. Epithelial cell damage was measured using a standard ^51^Cr release assay (29). Briefly, A549 epithelial cells were grown to 95% confluence in a 24-well tissue culture plate and loaded with ^51^Cr (ICN Biomedicals). After removal of the unincorporated ^51^Cr by rinsing, epithelial cells were infected with 5 × 10^5^ conidia in 1 ml of F-12 K medium per well. After incubation at 37°C in 5% (vol/vol) CO_2_ for 16 and 24 h, the medium covering the cells was collected. The cells were lysed with 6 N NaOH, and the wells were rinsed with Radiac wash (Biodex Medical Systems, Inc.). The lysate and rinses were combined, and the amount of ^51^Cr in the samples was determined by gamma counting. To measure the spontaneous release of ^51^Cr, uninfected A549 cells exposed to medium alone were processed in parallel. After adjusting for well-to-well differences in the incorporation of ^51^Cr, the percentage of speciﬁc release of ^51^Cr was calculated using the following formula: (experimental release − spontaneous release)/(total incorporation − spontaneous release).

### A549 endocytosis assay.

The epithelial cell endocytosis of the wild-type, Δ*ccpA*, and *ccpA*c strains was measured using a differential fluorescence assay as described previously (60). The three strains were transformed with a GFP expression plasmid containing the *ble* phleomycin resistance marker (GFP-Phleo) (61). Next, 10^7^ conidia of each strain were added to 20 ml Sabouraud dextrose broth in a Petri dish and incubated at 37°C for 4.5 h to produce swollen conidia. A549 pulmonary epithelial cells grown on fibronectin-coated glass coverslips in a 24-well tissue culture plate were infected with 10^5^ swollen conidia. After incubation for 3.5 h, the cells were washed with warm Hanks’ balanced salt solution (HBSS) in a standardized manner to remove nonadherent fungi and then fixed with 4% (vol/vol) paraformaldehyde for 15 min. The noninternalized organisms were sequentially stained with a polyclonal rabbit anti-*A. fumigatus* serum (Meridian Life Science, Inc.) and anti-rabbit antibody (Ab) conjugated with Alexa Fluor 568 (Life Technologies). Coverslips were mounted inverted on a microscope slide and observed under conditions of epifluorescence. The number of organisms endocytosed by host cells was determined by subtracting the number of noninternalized organisms (with red fluorescence) from the total number of organisms (with green fluorescence). At least 100 organisms were counted on each coverslip.

### Mouse infection models.

Established murine models for invasive pulmonary aspergillosis were used for virulence studies (62, 63). Female outbred CD-1 mice (Charles River) (18 to 20 g, 6 to 8 weeks old) were housed under standard conditions in individually ventilated cages and fed with normal mouse chow and water *ad libitum*.

### Cyclophosphamide model.

To induce neutropenia, cyclophosphamide was injected intraperitoneally (Sigma-Aldrich) (150 mg/kg) on days −4, −1, 2, 5, 8, and 11, with an additional subcutaneous dose of cortisone acetate (Sigma-Aldrich) (200 mg/kg) on day −1 prior to infection (day 0). Mice were anesthetized by an intraperitoneal anesthetic combination of midazolam, fentanyl, and medetomidine. For infection, 2 × 10^5^ conidia in 20 µl phosphate-buffered saline (PBS) were applied intranasally to the mice.

### Cortisone acetate (nonneutropenic) model.

Mice were immunosuppressed with two single doses of 25 mg cortisone acetate (Sigma-Aldrich), which were injected intraperitoneally 3 days before and immediately prior to infection (day 0). Mice were anesthetized as described above and intranasally infected with 1 × 10^6^ conidia in 20 µl PBS. Anesthesia was terminated by subcutaneous injection of flumazenil, naloxone, and atipamezol. Infected animals were monitored at least twice daily and humanely sacrificed if moribund (defined by severe lethargy, severe dyspnea, hypothermia, or substantial weight loss). Infections were performed with a group of 10 mice for each tested strain. A control group of 5 mice was mock infected (with PBS). For histopathological analyses, lungs from sacrificed animals were removed, fixed in formalin, and embedded in paraffin according to standard protocols. Sections (4 µm) were treated with periodic acid-Schiff stain (PAS) using standard protocols. The sections were analyzed with a Zeiss Axio Imager M2 microscope (Zeiss). Images were taken with an AxioCam 105 color microscope camera and analyzed by the use of AxioVision SE64 rel. 4.9.1 imaging software (Zeiss).

### Stimulation of human T cells.

The response of peripheral CD4^+^ cells to the recombinant CcpA protein was analyzed. Buffy coats or peripheral EDTA blood samples were obtained from the DRK Dresden, Dresden, Germany; from the Charité blood bank, Charité Berlin, Berlin, Germany; or from in-house volunteers. Peripheral blood mononuclear cells (PBMCs) from healthy donors were stimulated in RPMI 1640 (Gibco Life Technologies) supplemented with 5% (vol/vol) human AB serum (BioWhittaker/Lonza) and with 20 µg/ml of recombinant CcpA or A. fumigatus resting conidia crude lysate as described in reference 34 for 5 h in the presence of 1 µg/ml CD40 pure antibody and 1 µg/ml CD28 pure antibody (Miltenyi Biotec) and for an additional 2 h in the presence of 1 µg/ml brefeldin A (Sigma-Aldrich). Antigen-reactive T cell enrichment (ARTE) of reactive CD154^+^ Tcon cells was performed according to the protocols described in references 33 and 34. In brief, cells were indirectly magnetically labeled with CD154-biotin and anti-biotin microbeads and then enriched by the use of two sequential MS columns (Miltenyi Biotec). Reactive CD154^+^ T cells were counterstained for phenotypic and functional markers. The following monoclonal antibodies (MAbs) were used according to the manufacturers’ instructions: CD8-VioGreen (BW135/80), CD14-VioGreen (TÜK4), CD20-VioGreen (LT20), CD4-APC-Vio770 (VIT4), CD154-FITC (5C8), IL-17A-PE-Vio770 (CZ8-23G1), IL-10-APC (JES3-9D7) (from Miltenyi Biotec), CCR7-PE-Dazzle594 (G043H7), IFN-γ-PerCp-Cy5.5 (4S.B3), IL-2-BV605 (MQ1-17H12), and CD45RA-PE-Cy5 (HI100) (from BioLegend). Data were acquired on a BD LSR Fortessa analyzer (BD Biosciences) and analyzed using FlowJo software (Tree Star, Inc.).

### Statistical analysis.

Survival data were plotted as Kaplan-Meier curves and statistically analyzed by a log rank test using GraphPad Prism software 5.0 (GraphPad Software). The Student’s *t* test was used for significance testing of two groups. Differences between the groups were considered significant at a *P* value of ≤0.05 or ≤0.01. Throughout the article, significance is denoted as follows: *, *P* = <0.05; **, *P* = <0.01, ***, *P* = <0.001; ns, nonsignificant.

### Ethics statement.

For PMN experiments, human peripheral blood was collected from healthy volunteers after written informed consent was provided. The study was conducted in accordance with the Declaration of Helsinki and approved by the Ethics Committee of the University Hospital Jena (permit number 273-12/09). For DC experiments, ethical approval was obtained by the Ethical Committee of the University Hospital, Würzburg, for the use of blood of healthy donors (approval 34/15). Written informed consent was provided by all study participants. For T cell experiments, all donors gave consent and all protocols were approved by the Ethics Committee Charité (EA1/149/12). Mice were cared for in accordance with the principles outlined by the European Convention for the Protection of Vertebrate Animals Used for Experimental and Other Scientific Purposes (European Treaty Series, number 123; http://conventions.coe.int/Treaty/en/Treaties/Html/123.htm). All animal experiments were in compliance with the German animal protection law and were approved by the responsible Federal State authority “Thüringer Landesamt für Verbraucherschutz” and ethics committee “Beratende Komission nach §15 Abs. 1 Tierschutzgesetz” with permit number 03-001/12.
