# Supplementary figures and images for: Proteome Analysis Reveals the Conidial Surface Protein CcpA Essential for Virulence of the Pathogenic Fungus Aspergillus fumigatus
Source: mBio. 2018 Oct 2;9(5):e01557-18. doi: 10.1128/mBio.01557-18 (PMC6168859; doi:10.1128/mBio.01557-18)

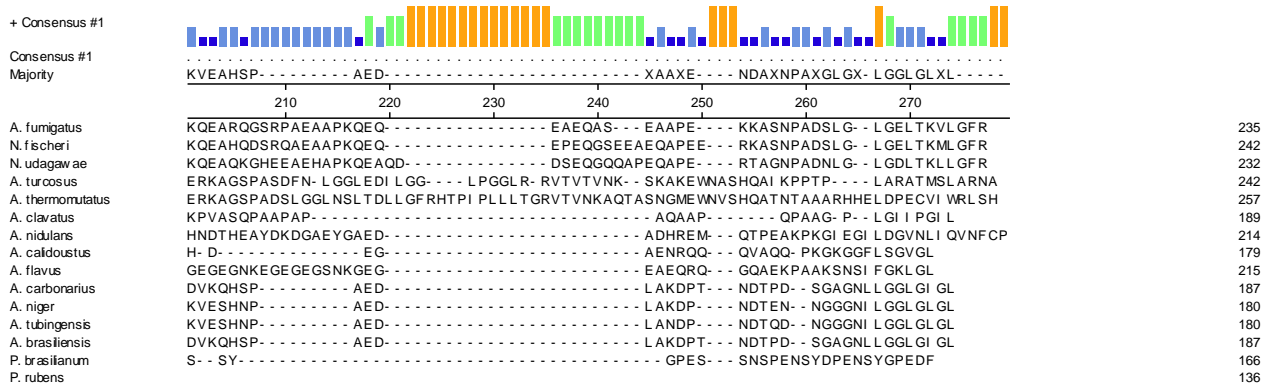

Supplement: FIG S1 [file mbo004184034sf1.pdf]

A

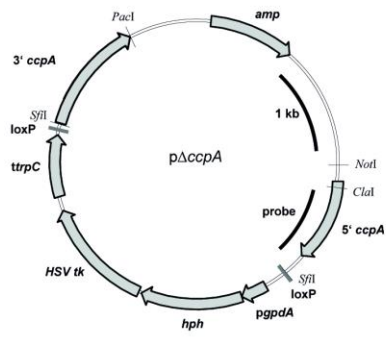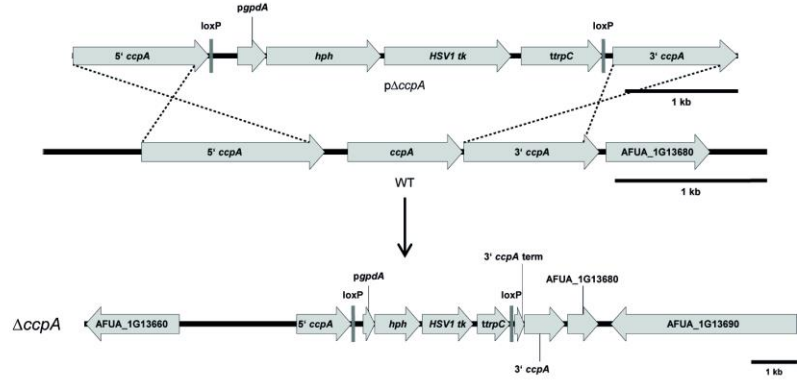

B

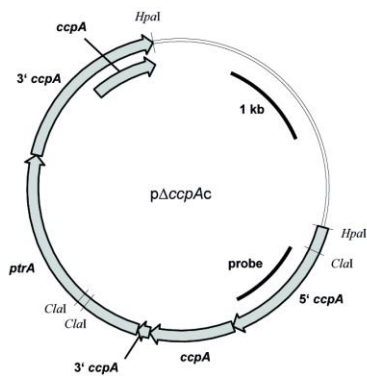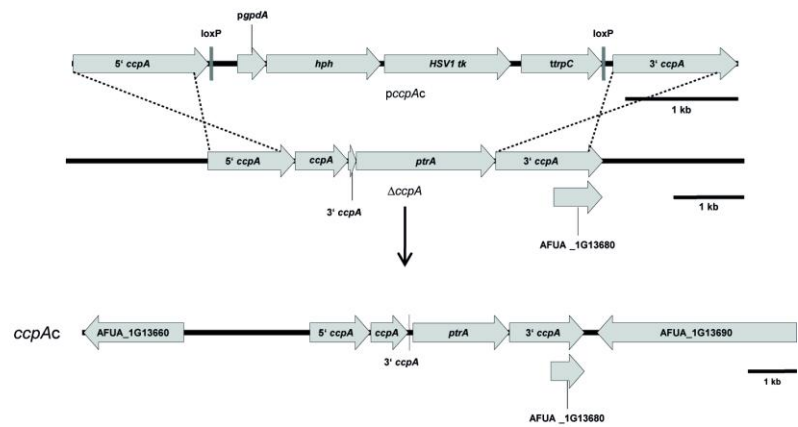

C

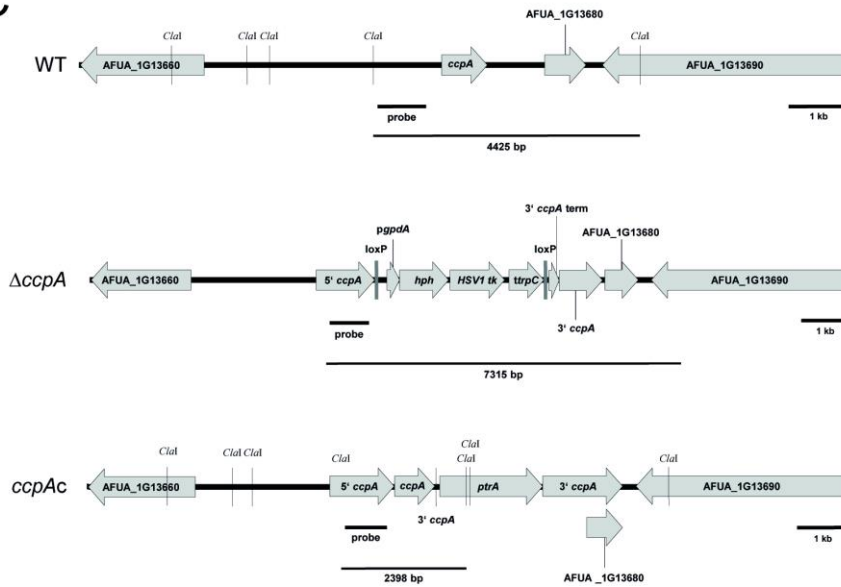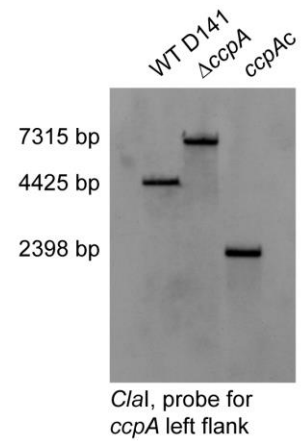

Supplement: FIG S2 [file mbo004184034sf2.pdf]

**A**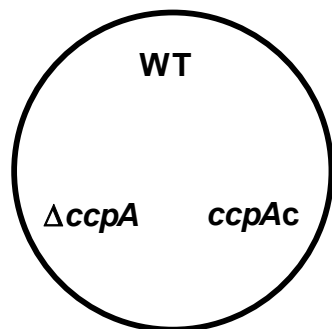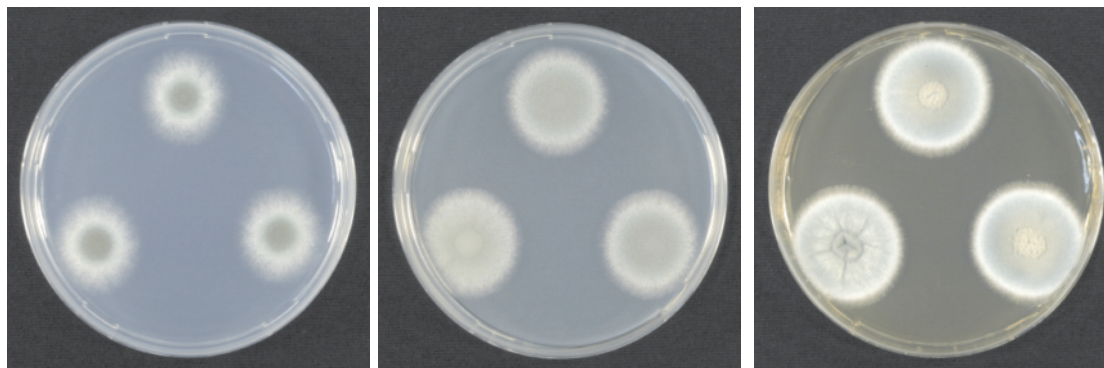**B**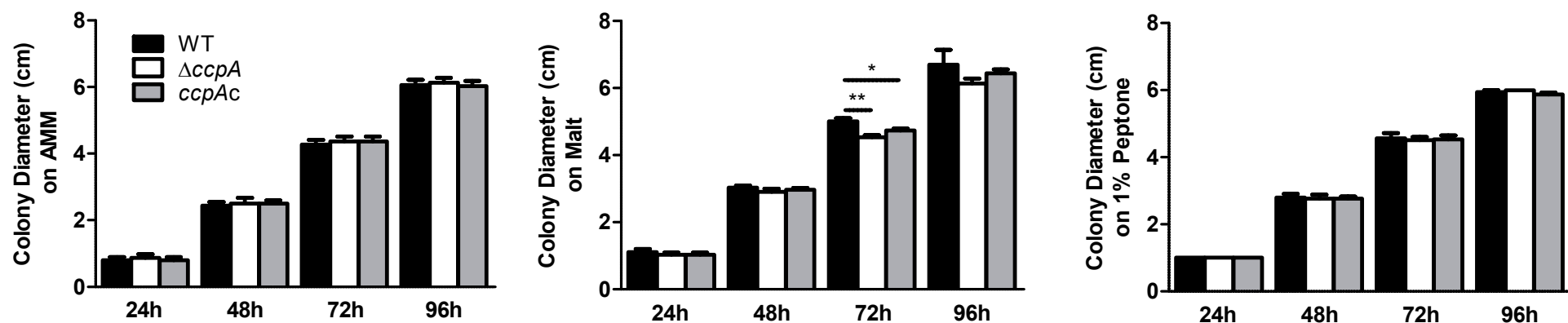**C**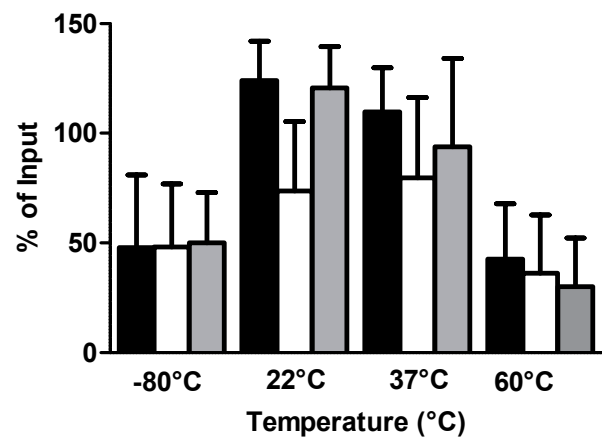**D**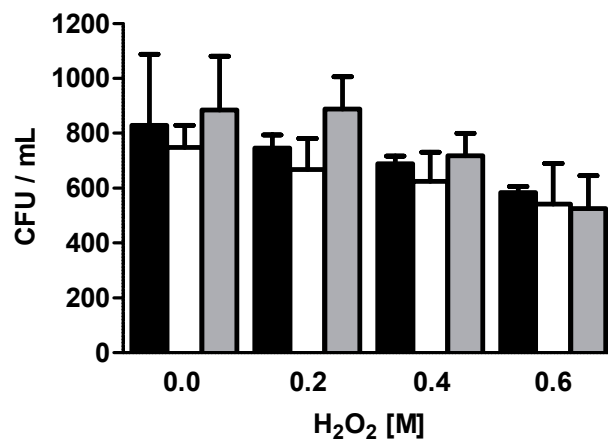

Supplement: FIG S3 [file mbo004184034sf3.pdf]

**A**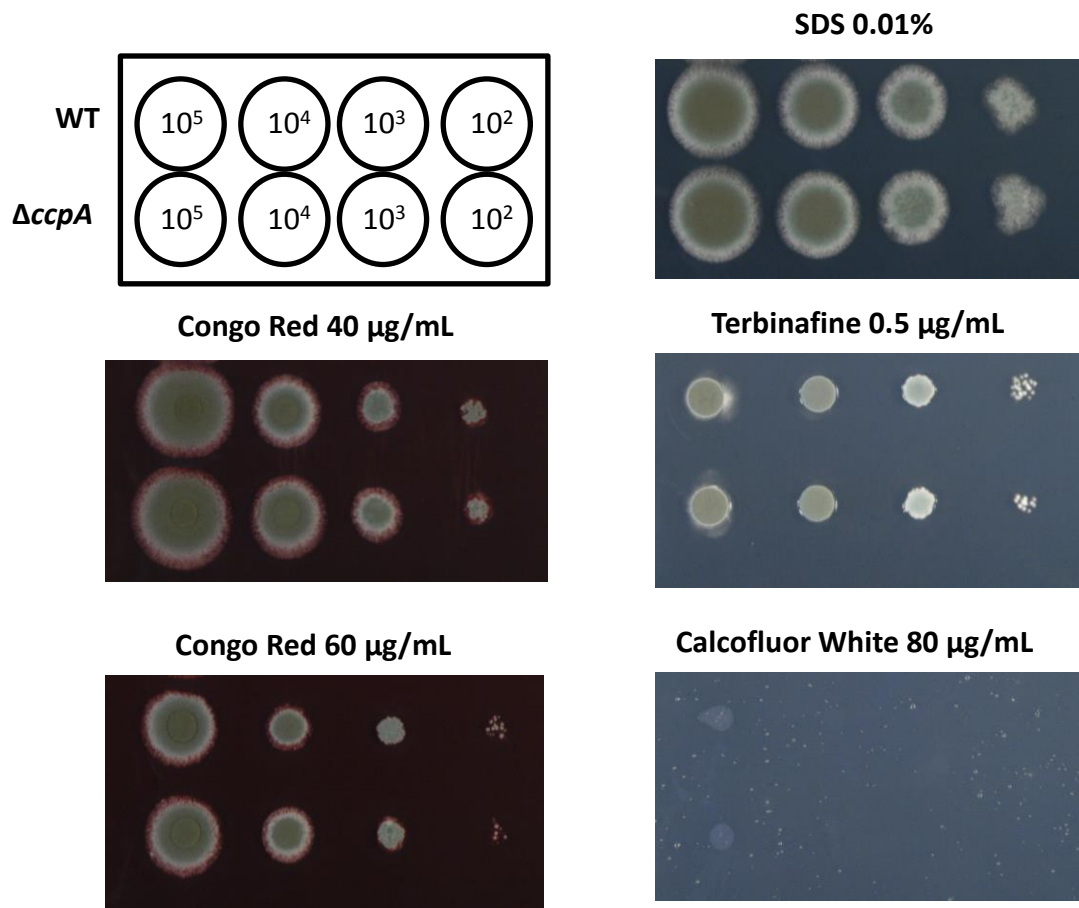**B****Wild type** **$\Delta ccpA$** 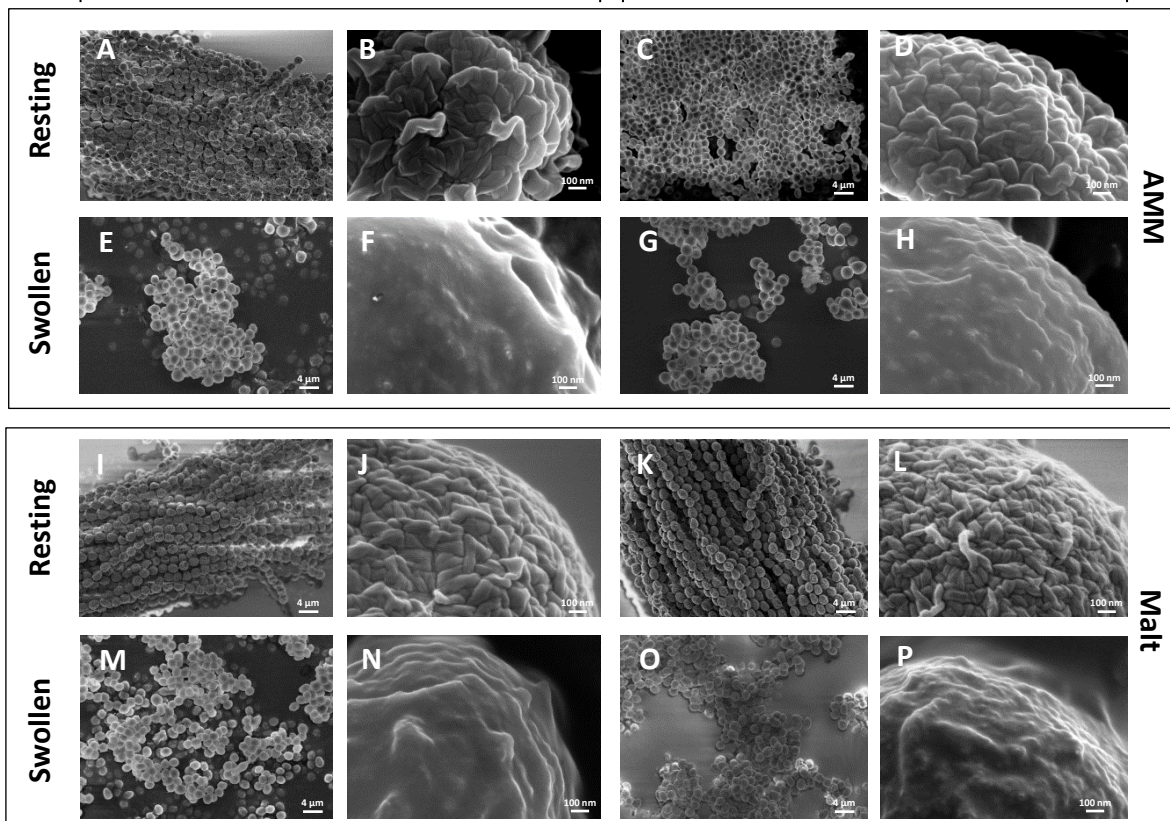

Supplement: FIG S4 [file mbo004184034sf4.pdf]

**A** AMM Resting

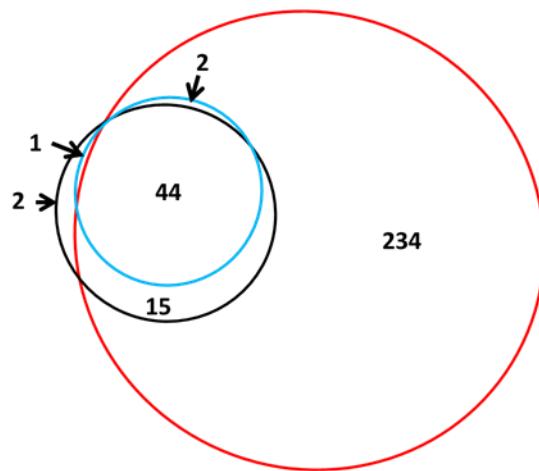

**B** Malt Resting

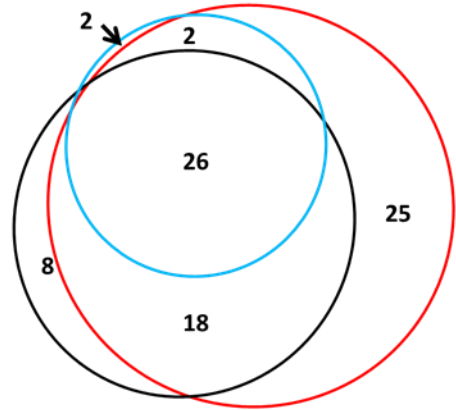

**C** AMM Swollen

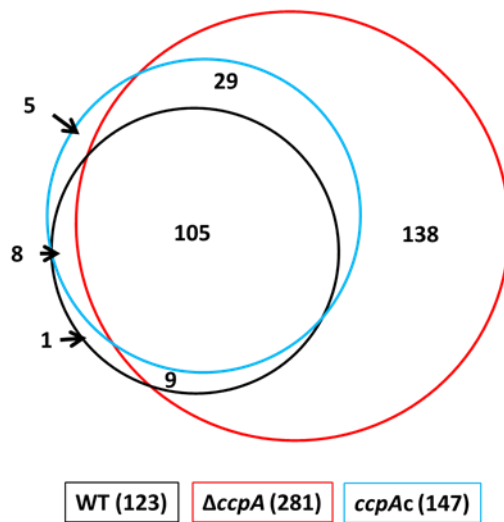

**D** Malt Swollen

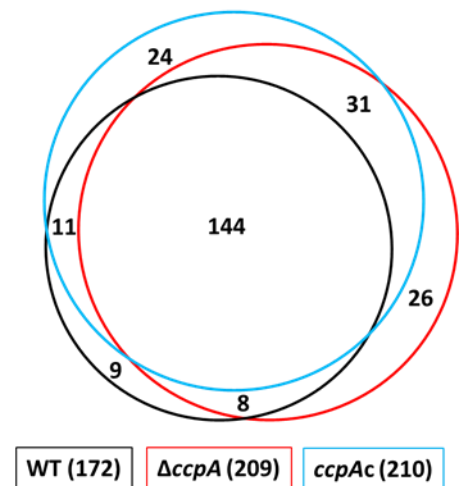

Supplement: FIG S6 [file mbo004184034sf6.pdf]

**A**

**WT**

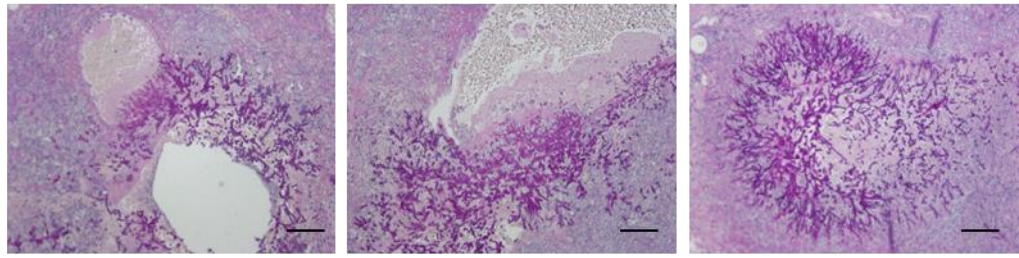

**$\Delta ccrA$**

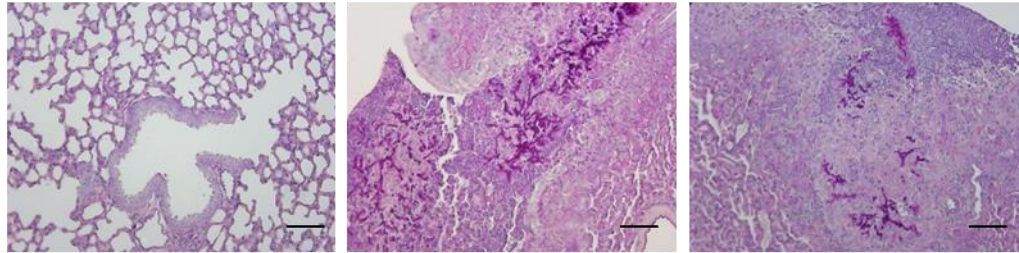

***ccrAc***

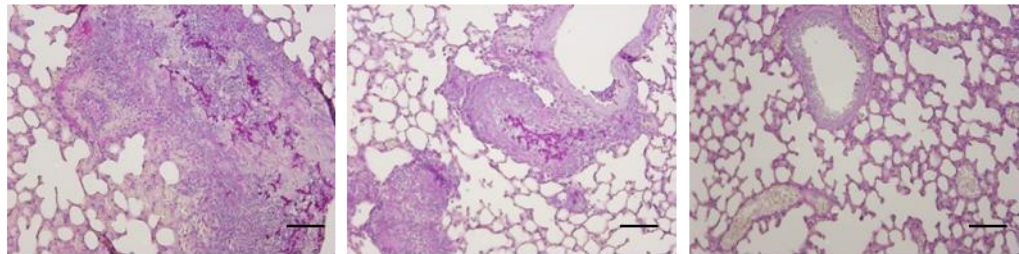

**B**

**PBS, 630 x**

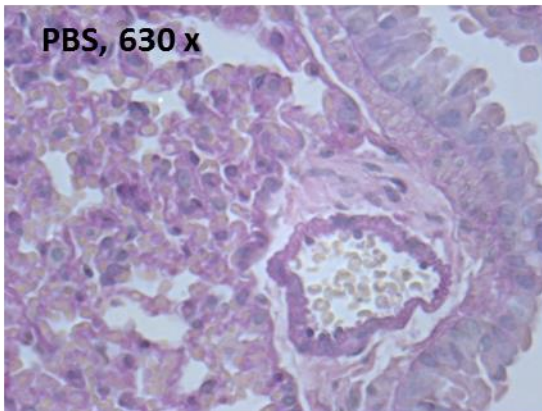

**WT, 3 d 630 x**

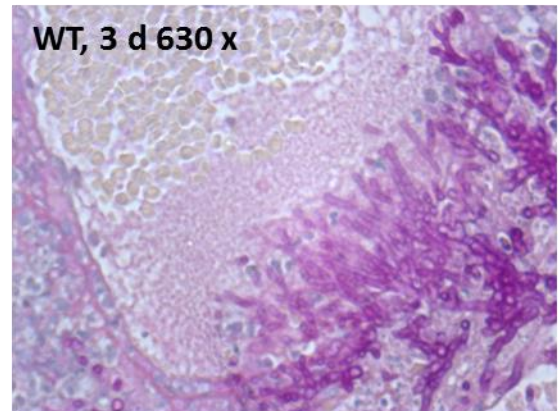

**$\Delta ccrA$ , 14 d 630x**

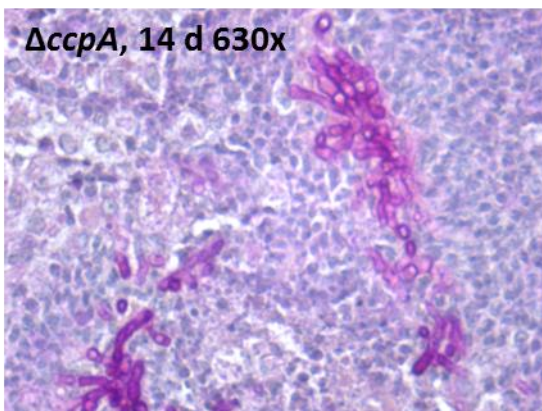

**WT, 6 d 630 x**

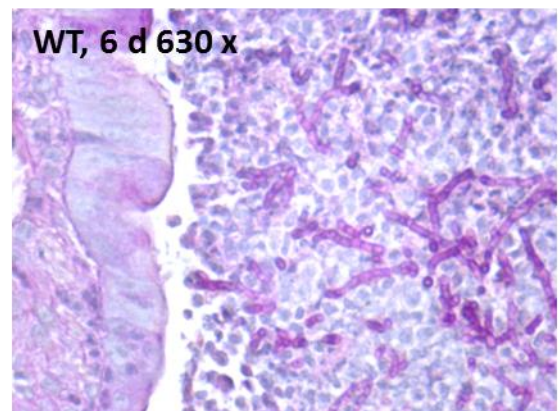

Supplement: FIG S7 [file mbo004184034sf7.pdf]

**A**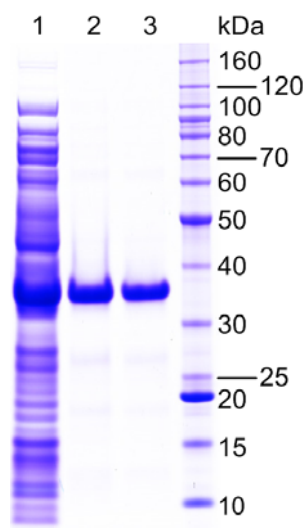**B**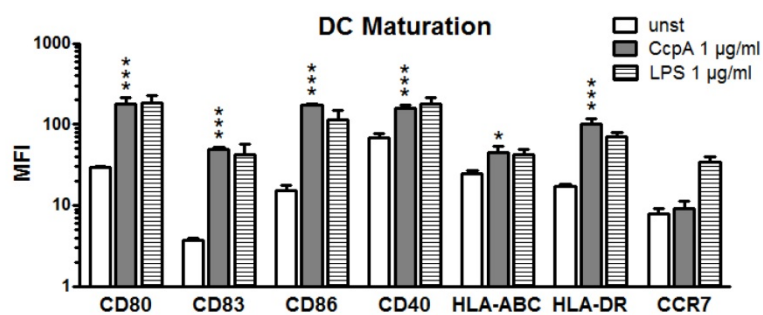**C**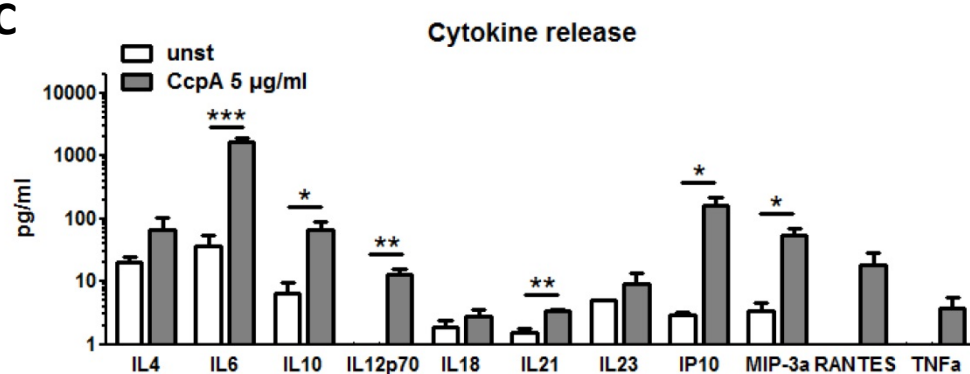

Supplement: FIG S8 [file mbo004184034sf8.pdf]
