# Supplementary material for: Proteome Analysis Reveals the Conidial Surface Protein CcpA Essential for Virulence of the Pathogenic Fungus Aspergillus fumigatus
Source: mBio. 2018 Oct 2;9(5):e01557-18. doi: 10.1128/mBio.01557-18 (PMC6168859; doi:10.1128/mBio.01557-18)
Supplement: FIG S5 [file mbo004184034sf5.pdf]

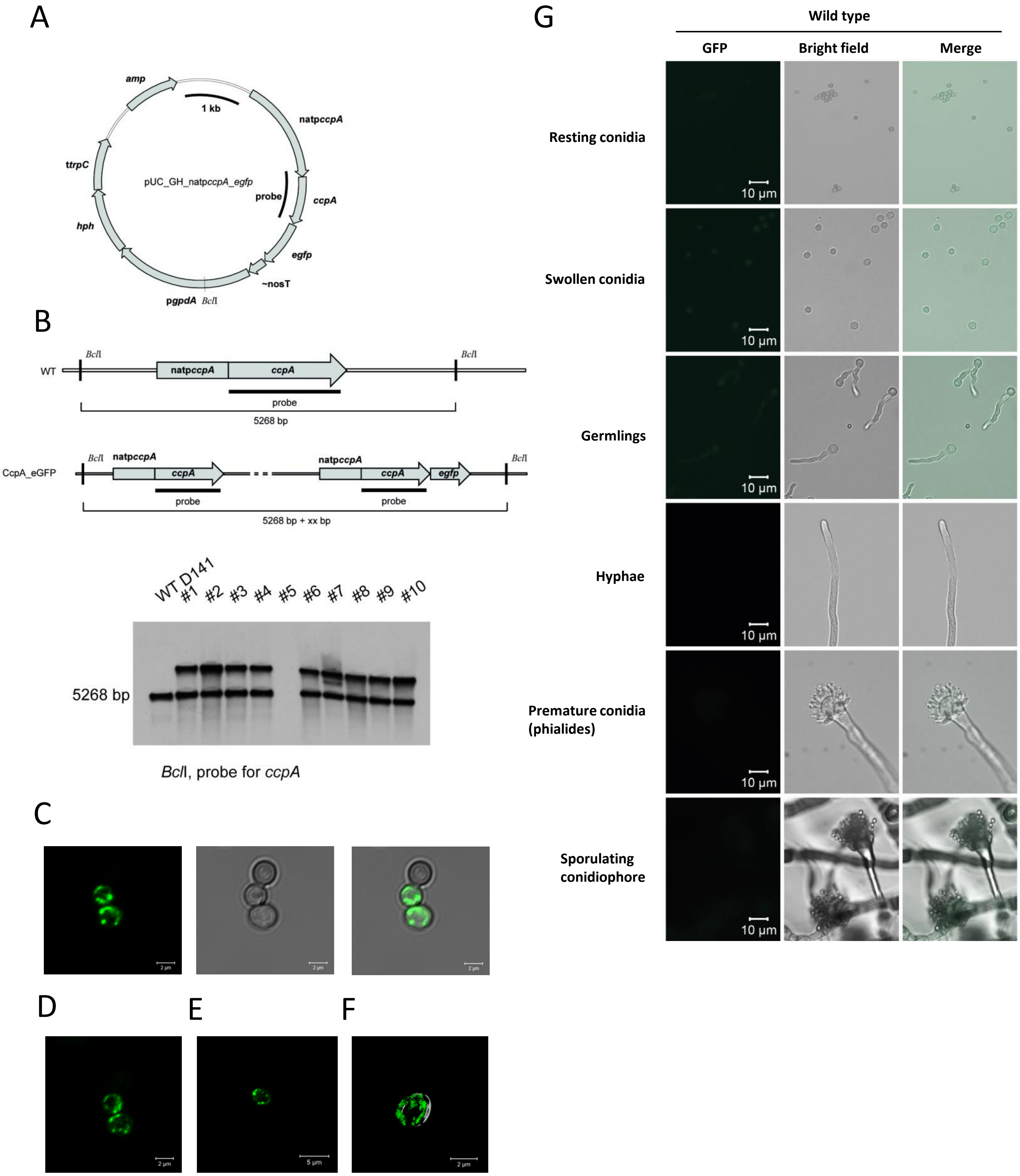
A
amp
1 kb
natpccpA
ttrpC
pUC_GH_natpccpA_egfp
probe
ccpA
hph
egfp
~nosT
pgpdA
BclI
B
WT
BclI
natpccpA
ccpA
BclI
probe
5268 bp
CcpA_eGFP
BclI
natpccpA
ccpA
natpccpA
ccpA
egfp
BclI
probe
probe
5268 bp + xx bp
WT D141
#1
#2
#3
#4
#5
#6
#7
#8
#9
#10
5268 bp
BclI, probe for ccpA
C
D
E
F
G
Wild type
GFP
Bright field
Merge
Resting conidia
10 µm
Swollen conidia
10 µm
Germlings
10 µm
Hyphae
10 µm
Premature conidia
(phialides)
10 µm
Sporulating
conidiophore
10 µm
