# Supplementary material for: Proteome Analysis Reveals the Conidial Surface Protein CcpA Essential for Virulence of the Pathogenic Fungus Aspergillus fumigatus
Source: mBio. 2018 Oct 2;9(5):e01557-18. doi: 10.1128/mBio.01557-18 (PMC6168859; doi:10.1128/mBio.01557-18)
Supplement: TABLE S2 [file mbo004184034st2.pdf]

S2 Table. Strains, plasmids, and oligonucleotides used in this study.

**Strain**	**Relevant Genotype/Phenotype**	**Reference**
ATCC 46645	*A. fumigatus* wild type	American Type Culture Collection
D141	*A. fumigatus* wild type, clinical isolate	[1]
Δ*ccpA*	derived from D141; *ccpA::hph*; Hyg^R, Δ*ccpA*	This study
*ccpA*c	derived from Δ*ccpA*; *ccpA*$^+$, *ptrA*; PT^R	This study
CcpA_eGFP	derived from D141; contains *ccpA_egfp*-fusion; construct under control of the native *ccpA*-promoter; Hyg^R	This study
Δ*ccpA*_CcpA_eGFP	derived from D141; contains a modified *ccpA_egfp*-fusion with *ptrA;* construct under control of the native *ccpA*-promoter; PT^R and Hyg^R	This study
*Af*PKSPEGFP4	derived from ATCC 46645; contains PpksP_egfp-fusion; construct under control of the native pksP-promoter; Hyg^R	[2]
E. coli α-Select		Bioline GmbH
E. coli BL21(DE3)		Agilent Technologies
E. coli BL21(DE3) pET28aHT*ccpA*_23-218	Kan^R	This study
**Plasmid**	**Genotype/Properties**	
pBlueskript II SK(+)_*Pac*I	general cloning plasmid with additional *Pac*I site	[3]
pSK275	Amp^R, PT^R (*ptrA*)	[4]
pSK397	*Sfi*I, loxP, p*gpdA*, Hyg^R (*hph*), *HSV1 tk*, t*trpC*, Amp^R (*amp*)	[5, 6]
pUC19L	linearised pUC19-Vector Amp^R (*amp*)	Invitrogen
pΔ*ccpA*	5'- and 3'-terminal flanking regions of *ccpA*, *loxP*, p*gpdA*, Hyg^R (*hph*), *HSV1 tk*, t*trpC*, Amp^R (*amp*)	This study
p*ccpA*c	5'- and 3'-terminal flanking regions of ccpA, PT^R (*ptrA*)	This study
pUC_GH	t*trpC*, p*gpdA*, p*otef*, *egfp*, Amp^R (*amp*), Hyg^R (*hph*)	[2]
pUC_GH_natp*ccpA_egfp*	natp*ccpA*, *ccpA*, *egfp*, p*gpdA*, Hyg^R (*hph*), t*trpC*, Amp^R (*amp*)	This study
pSK275_natp*ccpA_egfp*	natp*ccpA*, *ccpA*, *egfp*, Amp^R, PT^R (*ptrA*)	This study
pMAT_*ccpA*_23-218	synthetic gene, truncated *ccpA* (aa 23-218) cloned in pMal-derivative, MBP-tag, TEV site, Amp^R	Life Technologies
pET28aH6TEV	His_6-tag, TEV site, Kan^R (*kan*)	Novagen
pET28aHT*ccpA*_23-218	synthetic gene, truncated *ccpA* (aa 23-218) cloned in pET28-derivative, His_6-tag, TEV site, Kan^R	This study
**Oligonucleotide**	**Sequence (5' – 3')**	**Target Gene**
Deletion Plasmid		

ccpA-1	TATATGCGGCCGCTGGTGTAAGTCCATTTTCAAAGC	*ccpA*
ccpA-2	ATATTACCCGGGGGCCTGAGTGGCCTCATATGAGGACAACAATTCAACG	*ccpA*
ccpA-3	GAGTAGCCAGGCATGATTGC	*ccpA*
ccpA-4	CATCCTCAGGGACAGAGACC	*ccpA*
ccpA-5	ATATTAGAATTCGGCCATCTAGGCCAGTACTAAGCAGGACGCTCAACAG	*ccpA*
ccpA-6	TATCGTTAATTAACCCTCAGTTACTAGACCCGAAAG	*ccpA*
ccpA-7	TCTCGTTCCAGCTTGATTGC	*ccpA*
ccpA-8	AGTTCGCAGGAAGAGAGACG	*ccpA*
Complementation Plasmid		
sv831 (*ccpA*)	AATTCGAGCTCGGTACGTTAACTTGGTGTAAGTCCATTTTCAAAG	*ccpA*
sv832_*ptrA*_re (*ccpA*)	ATCCCGTAATCAATTAGAATACAAGACTGTGCGTATG	*ccpA*
sv833_*ptrA*_fw (*ccpA*)	AACAAAGATGCAAGATTAATAAGCACTTTTCCTTGACATG	*ccpA*
sv834 (*ccpA*)	GCCAAGCTTGCATGCCGTTAACCAAGAGAAGAATACCCTACAACC	*ccpA*
sv197 (ptrA)	AATTGATTACGGGATCCCATTGG	*ptrA*
sv198 (ptrA)	CATCTTTGTTTGTATTATACTGTCT	*ptrA*
Insert eGFP		
natP*ccpA*_fw_*Acc*65I	TGACCGGTACCCACTACAAGGGACCTTGACC	*ccpA*
*ccpA*_re_*Xma*I	CTTTGCCCGGGCCGGAAACCGAGCACCTTTGTG	*ccpA*
*ccpA*p_for_*KpnI*	ggtaccACTACAAGGGACCTTGACC	*ccpA-eGFP*
*nosT*_rev_*KpnI*	ggtaccTCGACGTATTTCAGTGTCG	*ccpA-eGFP*
*ccpA*p_for	CCAGGAACCCCGTTGATAAG	*ccpA-eGFP*
*nosT*_rev	GTTTGACAGCTTATCATCGG	*ccpA-eGFP*
Recombinant Protein		
*ccpA*_23*Bam*HIfw	AACTGGGATCCCGTCAGGGTGCAGCAGCATTTGTTA	*ccpA*
*ccpA*_218*Hin*dIIIre	ACTGAAGCTTTTAATTGCTTGCTTTTTTTTCCGGTGCGGC	*ccpA*
Hyg^R: Hygromycin-resistance; Kan^R: Kanamycin-resistance; PT^R: Pyrithiamine-resistance; *ptrA*: Pyrithiamine-resistance cassette; Amp^R: Ampicillin-resistance; MBP: Maltose-binding protein; p*gpdA*: *A. nidulans gpdA* promoter; t*trpC*: terminator region of the *A. nidulans trpC* gene; *HSV1 tk*: thymidine kinase-encoding sequence from the herpes simplex virus type 1; loxP: specific recombinase binding sites of plasmid pSK397; Restriction sites are underlined: *Sfi*I - dotted; *Not*I - solid; *Xma*I - double; *Eco*RI - bold; *Pac*I - wavy; *Acc*65I - bold-wavy; *Hpa*I - bold-dotted; *Bam*HI – dashed; *Hin*dIII – bold-dashed, *KpnI* – lowercase		

References

1. Reichard U, Büttner S, Eiffert H, Staib F, Rüchel R. Purification and characterisation of an extracellular serine proteinase from *Aspergillus fumigatus* and its detection in tissue. J Med Microbiol. 1990;33(4):243-51. PubMed PMID: 2258912.

2. Langfelder K, Philippe B, Jahn B, Latge JP, Brakhage AA. Differential expression of the *Aspergillus fumigatus pksP* gene detected *in vitro* and *in vivo* with green fluorescent protein. Infect Immun. 2001;69(10):6411-8. doi: 10.1128/IAI.69.10.6411-6418.2001. PubMed PMID: 11553585; PubMed Central PMCID: PMCPMC98776.

3. Sriranganadane D, Reichard U, Salamin K, Fratti M, Jousson O, Waridel P, et al. Secreted glutamic protease rescues aspartic protease Pep deficiency in *Aspergillus fumigatus* during growth in acidic protein medium. Microbiol. 2011;157(Pt 5):1541-50. doi: 10.1099/mic.0.048603-0. PubMed PMID: 21349972.

4. Szewczyk E, Krappmann S. Conserved regulators of mating are essential for *Aspergillus fumigatus* cleistothecium formation. Eukaryot Cell. 2010;9(5):774-83. doi: 10.1128/EC.00375-09. PubMed PMID: 20348388; PubMed Central PMCID: PMCPMC2863953.

5. Krappmann S, Bayram O, Braus GH. Deletion and allelic exchange of the *Aspergillus fumigatus veA* locus via a novel recyclable marker module. Eukaryot cell. 2005;4(7):1298-307. PubMed PMID: 16002655.

6. Krappmann S, Sasse C, Braus GH. Gene targeting in *Aspergillus fumigatus* by homologous recombination is facilitated in a nonhomologous end- joining-deficient genetic background. Eukaryot Cell. 2006;5(1):212-5. Epub 2006/01/10. doi: 10.1128/ec.5.1.212-215.2006. PubMed PMID: 16400185; PubMed Central PMCID: PMC1360265.
